# Mechanochemical Dimerization of Aldoximes to Furoxans

**DOI:** 10.3390/molecules27082604

**Published:** 2022-04-18

**Authors:** Run-Kai Fang, Kuan Chen, Chuang Niu, Guan-Wu Wang

**Affiliations:** 1 Hefei National Laboratory for Physical Sciences, Microscale and Department of Chemistry, University of Science and Technology of China, Hefei 230026, China; frk4321@mail.ustc.edu.cn (R.-K.F.); kuanc@mail.ustc.edu.cn (K.C.); cniu@mail.ustc.edu.cn (C.N.); 2 State Key Laboratory of Applied Organic Chemistry, Lanzhou University, Lanzhou 730000, China

**Keywords:** mechanochemistry, aldoximes, furoxans, nitrile oxides, dimerization

## Abstract

Solvent-free mechanical milling is a new, environmentally friendly and cost-effective technology that is now widely used in the field of organic synthesis. The mechanochemical solvent-free synthesis of furoxans from aldoximes was achieved through dimerization of the in situ generated nitrile oxides in the presence of sodium chloride, Oxone and a base. A variety of furoxans was obtained with up to a 92% yield. The present protocol has the advantages of high reaction efficiency and mild reaction conditions.

## 1. Introduction

The furoxans (1,2,5-oxadiazole 2-oxides) are an important class of heterocyclic compounds with a long history [1]. Because of their ability to release NO [2,3], they play an important role in biochemistry, pharmaceuticals and other fields [4,5,6,7,8]. Over the past few decades, extensive work has been devoted to their synthesis. Among them, the commonly used approaches are oxidation of aldoximes, dehydration of nitrobenzenes and pyrolysis of *o*-nitroazidobenzenes [9,10,11,12,13,14,15,16,17,18]. In general, most of these preparation methods are very useful, but they often suffer from some drawbacks, such as the use of complicated starting materials, special oxidants, toxic organic solvents and so on. Therefore, it is necessary to develop an efficient and environmentally friendly method to synthesize furoxans from readily available starting materials.

Solvent-free reactions have drawn much attention in recent years. As one attractive type of solvent-free reactions mechanochemistry does not use organic solvents in the reaction process, or only a small amount of liquid-assisted grinding (LAG) is used, which can greatly reduce waste discharge [19,20,21,22,23,24,25,26]. In addition, mechanochemical protocols have the advantages of shortening the reaction time, reducing the reaction temperature, improving reaction selectivity, and can even provide products that are difficult or impossible to access in liquid-phase reactions [27,28,29,30,31]. Therefore, solvent-free mechanochemical reactions have been effectively used in organic synthesis [32,33,34,35,36,37,38].

**Scheme 1:**
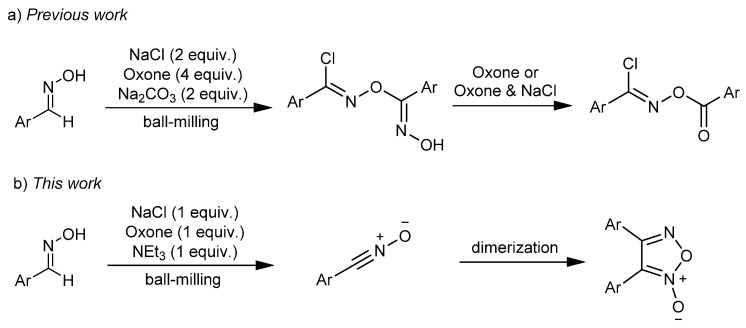
Comparison of different pathways in our previous and current work.

Aldoximes have drawn increasing attention in organic synthesis due to their easy availability, better selectivity and tolerance to various functional groups. Oxone (2KHSO_5_·KHSO_4_·K_2_SO_4_) is a stable and non-toxic inorganic oxidant, as demonstrated in reactions involving aldoximes [39,40,41,42]. Oxone has also been employed in mechanochemical reactions [42,43,44,45]. In 2019, the Tong group reported a protocol to generate nitrile oxides through NaCl/Oxone oxidation of aldoximes [41]. We previously reported the formation of *N*-acyloxyimidoyl chlorides from the mechanochemical solvent-free reaction of aldoximes with NaCl and Oxone in the presence of Na_2_CO_3_ (Scheme 1a) [42]. It was found that product distribution and product yield were sensitive to the molar ratio of the reagents as well as the employed base. Interestingly, certain amounts of furoxans could be generated from aldoximes under the modified conditions. To further study this new reaction, we decided to optimize the reaction conditions. To our satisfaction, a mild and efficient method was established for the formation of furoxans after detailed explorations (Scheme 1b).

## 2. Results and Discussion

Our initial investigation was started by using (*E*)-4-methylbenzaldehyde oxime (**1a**) as a representative substrate. A mixture of **1a** (0.2 mmol), NaCl (1.0 equiv.), Oxone (1.0 equiv.) and Na_2_CO_3_ (1.0 equiv.) together with four stainless-steel balls (5 mm in diameter) was introduced into a stainless-steel jar (5 mL) and milled (30 Hz) in a Retsch MM400 mixer mill (Retsch GmbH, Haan, Germany) at room temperature for 30 min. After separation, an 8% yield of **2a** was obtained (Table 1, entry 1). The product distribution was significantly affected by the choice of the used base. When Na_2_CO_3_ was replaced by other inorganic bases, including NaO*^t^*Bu, NaOAc and NaHCO_3_, only a trace amount of **2a** was obtained (Table 1, entries 2–4). Satisfyingly, the desired product **2a** was isolated in 36% yield when K_2_CO_3_ was employed as the base (Table 1, entry 5). However, Cs_2_CO_3_ afforded **2a** in only a 7% yield (Table 1, entry 6). Then, we tried to use organic bases, such as 4-dimethylaminopyridine (DMAP), 1,8-diazabicyclo[5.4.0]undec-7-ene (DBU) and 1,4-diazabicyclo[2.2.2]octane (DABCO), which did not afford the product at all (Table 1, entries 7–9). To our delight, when triethylamine (NEt_3_) was employed, **2a** could be obtained in a 79% yield (Table 1, entry 10). Based on this result, the influence of the amount of NEt_3_ on the product yield was investigated. The product yield was decreased to 69% and 53% when the amount of NEt_3_ was increased to 1.25 equiv. and 1.5 equiv., respectively (Table 1, entries 11 and 12), showing a detrimental effect of excess NEt_3_. The exact reason is unknown so far. On the other hand, the yield was reduced to 62% and 38% when the equivalent of NEt_3_ was less than the required stoichiometric amount (0.75 equiv. and 0.5 equiv., respectively) (Table 1, entries 13 and 14). When a mixture of **1a** (0.2 mmol), NaCl (1.0 equiv.), Oxone (1.0 equiv.) and NEt_3_ (1.0 equiv.) was magnetically stirred at room temperature for 2 h, only a trace amount of **2a** could be obtained (Table 1, entry 15). This result demonstrated the great advantage of the current reaction by ball milling over magnetic stirring. Then, the reaction time was investigated. When the reaction time was shortened from 30 min to 15 min, the desired product **2a** was obtained in only a 47% yield (Table 1, entry 16). When prolonging the reaction time to 40 min, the yield had no further improvement (Table 1, entry 17). When the amounts of NaCl and Oxone were increased, the yield essentially remained the same (Table 1, entries 18 and 19). The LAG protocol has been shown to improve reaction efficiency in mechanochemical reactions [31,32,46,47,48,49,50]. Accordingly, several liquids were added to the reaction mixture as LAG agents. However, ethyl alcohol (EtOH), dichloromethane (DCM), ethyl acetate (EtOAc) and acetonitrile (CH_3_CN) were detrimental to the reaction, and **2a** were obtained in 50–71% yields (Table 1, entries 20–23). Therefore, optimized reaction conditions were established as follows: 0.2 mmol of **1a**, 1.0 equiv. of NaCl, 1.0 equiv. of Oxone and 1.0 equiv. of NEt_3_ at 30 Hz for 30 min (Table 1, entry 10).

**Scheme 2:**
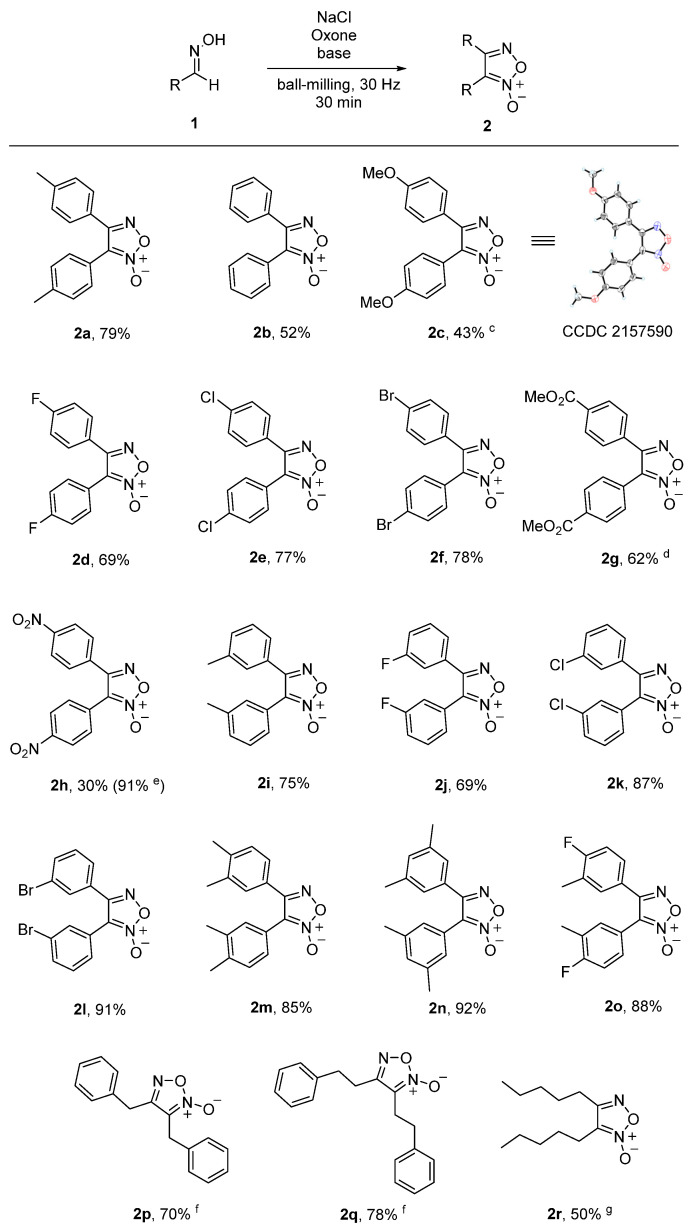
Scope of aldoximes (**1a**–**r**) ^a,b^. ^a^ Unless otherwise stated, the reactions were performed in a stainless-steel jar (5 mL) with (**1a**–**r**) (0.2 mmol), NaCl (0.2 mmol), Oxone (0.2 mmol), NEt_3_ (0.2 mmol) together with four stainless-steel balls (5 mm in diameter) using a Retsch MM400 mixer mill at 30 Hz for 30 min. ^b^ Isolated yields based on (**1a**–**r**). ^c^ NaCl (0.8 mmol), Oxone (0.2 mmol), Na_2_CO_3_ (0.4 mmol). ^d^ Reaction time was 60 min. ^e^ NaCl (0.4 mmol), Oxone (0.6 mmol), NaO*^t^*Bu (0.2 mmol). ^f^ NaCl (0.4 mmol), Oxone (0.2 mmol), Na_2_CO_3_ (0.4 mmol). ^g^ NaCl (0.4 mmol), Oxone (0.2 mmol), Na_2_CO_3_ (0.6 mmol).

With the optimized reaction conditions in hand, the scope and generality of this reaction were then examined, and the results are shown in Scheme 2. At first, a variety of aromatic aldoximes (**1a**–**o**) were explored and found to be compatible under the optimized reaction conditions. The substrate **1b** with no substituent on the phenyl ring gave the corresponding product **2b** in a 52% yield. For the aldoxime **1c** containing the strong electron-rich *para*-OMe group, very low efficiency was observed with NEt_3_ as a base. To our delight, product **2c** could be obtained in a 43% yield when NEt_3_ was replaced by Na_2_CO_3_. As for the *para*-halogen-substituted (*E*)-benzaldehyde oximes **1d**–**f**, the desired products **2d**–**f** were synthesized in 69–78% yields. For the substrate **1g** with the *para*-substituted CO_2_Me group, the corresponding product **2g** was isolated in a 62% yield after prolonging the reaction time to 60 min. When the aldoxime **1h** bearing the strong electron-deficient *para*-NO_2_ was investigated, the desired product **2h** was obtained in only a 30% yield. However, NaO*^t^*Bu could replace NEt_3_ to achieve a high yield of 91% for product **2h**. As for the *meta*-substituted substrates **1i**–**l** bearing Me, F, Cl and Br, the desired products **2i**–**l** were isolated in 69–91% yields. The disubstituted substrates **1m**–**o** were also compatible under the standard reaction conditions, affording products **2m**–**o** in good yields of 85–92%. Unfortunately, it was found that heteroaromatic aldoximes, including (*E*)-nicotinaldehyde oxime, (*E*)-thiophene-2-carbaldehyde oxime and (*E*)-benzofuran-2-carbaldehyde oxime, were not suitable substrates for the current reaction. To further illustrate the substrate scope of this reaction, the substrates were extended from the aromatic aldoximes to aliphatic aldoximes with Na_2_CO_3_ as the base. Gratifyingly, the (*E*)-2-phenylacetaldehyde oxime **1p** gave the corresponding product **2p** in a 70% yield. When (*E*)-3-phenylpropanal oxime **1q** was employed under the newly modified reaction conditions, the desired **2q** was obtained in a 78% yield. Another aliphatic aldoxime **1r** formed from hexanal was also applicable to our reaction and provided **2r** in a 50% yield. The structures of products were unambiguously confirmed by single-crystal X-ray diffraction analysis with **2c** as an example (see the Supplementary Materials for details).

It is noteworthy that NEt_3_ was used as the base for the efficient formation of furoxans in most cases. For the strong electron-deficient aldoxime **1h** bearing 4-NO_2_Ph group, a stronger base NaO*^t^*Bu could dramatically increase the product yield. In contrast, for the electron-rich substrates including aldoxime **1c** containing the 4-OMePh group and aliphatic aldoximes **1p**–**r**, a weaker base Na_2_CO_3_ was required. The exact reasons for these phenomena are not yet clear but it is likely that the different basicity of the employed three bases matches the formation of the corresponding 1,3-dipolar nitrile oxides and the subsequent dimerization.

**Scheme 3:**
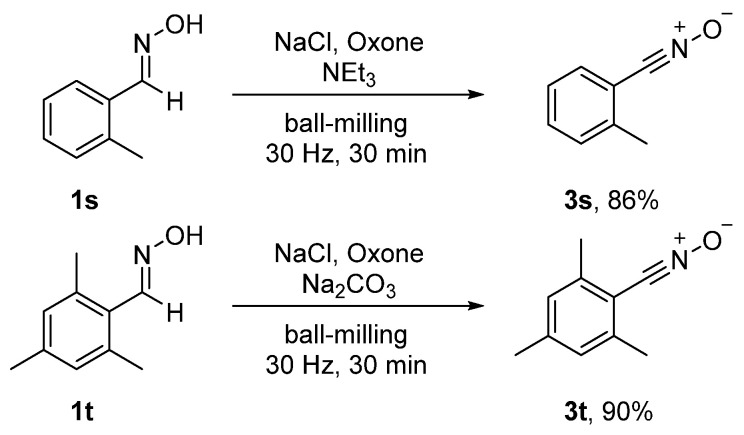
Formation of nitrile oxides from **1s** and **1t** under ball-milling conditions.

During the course of our studies, we tried to use the *ortho*-substituted (*E*)-2-methylbenzaldehyde oxime (**1s**) as the substrate. Intriguingly, the nitrile oxide **3s** rather than its dimer was obtained in an 86% yield, probably due to the steric hindrance caused by the *ortho*-substituent. Similarly, the 1,3-dipole **3t** was isolated in a 90% yield when (*E*)-2,4,6-trimethylbenzaldehyde oxime (**1t**) was employed (Scheme 3).

**Scheme 4:**
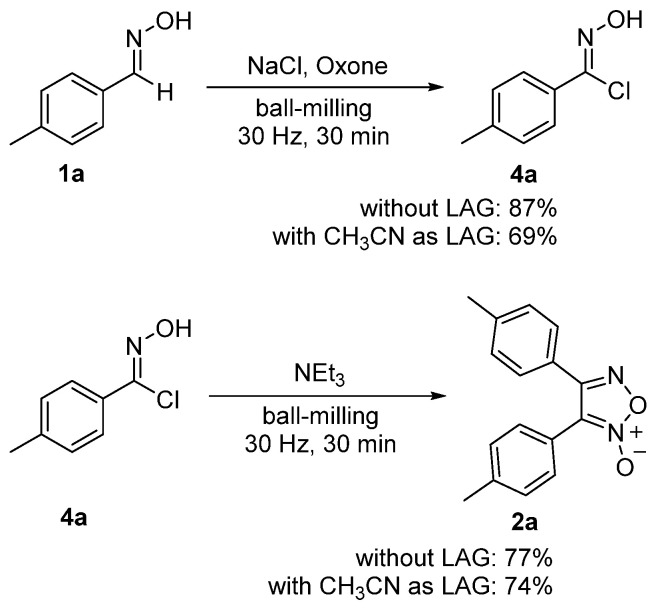
Control experiments.

To gain insight into the reaction mechanism of this transformation, control experiments were performed (Scheme 4). The reaction of **1a** (0.2 mmol), NaCl (1 equiv.) and Oxone (1 equiv.) afforded hydroximoyl chloride **4a** in an 87% yield under our solvent-free ball-milling conditions. Then, **4a** was allowed to react with NEt_3_ (1 equiv.) under the ball-milling conditions and produced **2a** in a 77% yield. The effect of LAG on these two reactions was also examined. It was found that CH_3_CN as LAG seemed to retard the formation of **4a** to a certain degree and showed nearly no effect on the subsequent dimerization process. Thus, these control experiments demonstrated that **4a** should be the key intermediate for the transformation to **2a** and explained why a slightly overall lower yield for the formation of **2a** was observed with CH_3_CN as the LAG agent (71% vs. 79%, Table 1, entry 23 vs. entry 10).

**Scheme 5:**
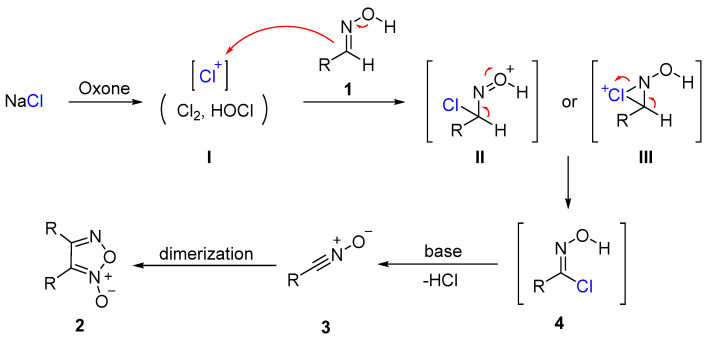
Proposed mechanism for the formation of **2**.

On the basis of the above experimental results and previous literature [41,42], a plausible mechanism is proposed (Scheme 5). First, NaCl is oxidized by Oxone to generate the chlorinating species **I**. Then, aldoxime **1** undergoes a chlorination reaction with **I** to provide the hydroximoyl chloride **4** via the possible intermediate **II** or **III**. Subsequently, **4** eliminates HCl with the aid of base, and the resulting nitrile oxide **3** undergoes 1,3-dipolar addition to the C≡N bond of another **3** to give the dimerization product **2**.

**Scheme 6:**
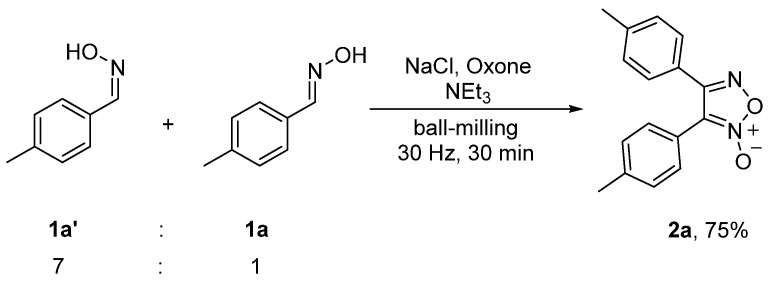
Mechanosynthesis of **2a** from a mixture of **1a** and **1a′**.

The aldoximes used in the above experiments were prepared according to the reported procedure [41] and were determined as the *E*-isomers [51]. The *E*-isomers of aldoximes could be converted into the corresponding *Z*-isomers under acidic conditions [51]. When a mixture of (*Z*)-4-methylbenzaldehyde oxime (**1a’**) and the *E*-isomer **1a** in a molar ratio of 7:1 was employed to replace the single isomer **1a**, **2a** was isolated in a 75% yield (Scheme 6), indicating that both *E*- and *Z*-isomers of aldoximes could provide furoxans in essentially the same yields.

**Scheme 7:**
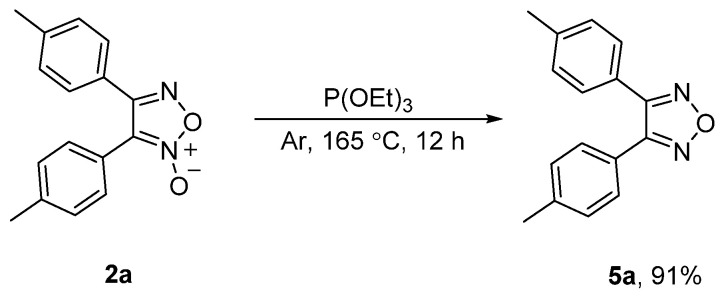
The deoxygenation reaction of **2a**.

To demonstrate the utility of the obtained furoxans, **2a** could be deoxygenated by triethyl phosphite (P(OEt)_3_) to provide 1,2,5-oxadiazole **5a** in 91% yield at 165 °C for 12 h under an argon atmosphere (Scheme 7) [52].

For the purpose of comparing the present solvent-free reaction with its liquid-phase counterpart, we performed the reaction of **1a** (0.2 mmol) with NaCl (1.0 equiv.), Oxone (1.0 equiv.) and NEt_3_ (1.0 equiv.) in several organic solvents including dimethyl sulfoxide (DMSO), *N*,*N*-dimethylformamide (DMF), acetonitrile (MeCN) and 1,2-dichloroethane (DCE) at room temperature for 2 h. The results showed that MeCN was the best solvent, and the yield of product **2a** was 47%. It is obvious that the present mechanochemical protocol has the higher yield (79% vs. 47%) and shorter reaction time (30 min vs. 120 min) under solvent-free conditions compared to the liquid-phase counterpart reaction. The possible reason is that the possibility of close contact of 1,3-dipoles for dimerization under the solvent-free mechanical milling conditions is much higher than that in the liquid phase.

Green chemistry metrics, such as complete and simple environmental factors (cEF and sEF), atom economy (AE) and reaction mass efficiency (RME), for the mechanosynthesis of **2a** were quantified, showing advantages in greenness compared with those of its liquid-phase counterpart (see the Supplementary Materials for details).

## 3. Materials and Methods

### 3.1. General Information

All reagents were obtained from commercial sources and used without further purification. NMR spectra were recorded on a Bruker Advance III HD 400 NMR spectrometer (Bruker BioSpin AG, Fällanden, Switzerland; 400 MHz for ^1^H NMR; 101 MHz for ^13^C NMR; 376 MHz for ^19^F NMR) and a Bruker Advance III HD 500 NMR spectrometer (Bruker BioSpin AG, Fällanden, Switzerland; 500 MHz for ^1^H NMR; 126 MHz for ^13^C NMR; 471 MHz for ^19^F NMR). ^1^H NMR chemical shifts were determined relative to TMS at 0.00 ppm, CDCl_3_ at *δ* 7.26 ppm or DMSO-*d*_6_ at *δ* 2.50 ppm. ^13^C NMR chemical shifts were determined relative to TMS at 0.00 ppm, CDCl_3_ at *δ* 77.16 ppm or DMSO-*d*_6_ at *δ* 39.52 ppm. Data for ^1^H NMR and ^13^C NMR are reported as follows: chemical shift (*δ*, ppm), multiplicity (s = singlet, d = doublet, t = triplet, m = multiplet). High-resolution mass spectra (HRMS) were taken on a Waters Acquity UPLC-Xevo G2 QTof mass spectrometer (Waters, Milford, MA, USA) with FTMS-ESI in positive mode. Ball-milling reactions were performed in a MM400 mixer mill (Retsch GmbH, Haan, Germany), using a 5 mL stainless-steel jar with four 5 mm diameter stainless-steel balls and were milled at a frequency of 1800 rounds per minute (30 Hz) at room temperature. *E*-Aldoximes **1** were prepared according to the reported protocol [41]. A mixture of *Z*-isomer **1a’** and *E*-isomer **1a** was obtained in molar ratio of 7:1 by stirring *E*-**1a** in trifluoroacetic acid (TFA)-CHCl_3_ at 0 °C for 20 min followed by removal of the volatiles in vacuo [51]. Single crystals of **2c** were grown from dichloromethane/*n*-hexane at 4 °C.

### 3.2. Mechanochemical Synthesis and Characterization of Products ***2a***–***r***, ***3s*** and ***3t***

A mixture of aldoximes **1a**–**t** (0.2 mmol), NaCl (0.2 mmol), Oxone (0.2 mmol) and base (0.2 mmol) together with four stainless-steel balls (5 mm in diameter) was introduced into a stainless-steel jar (5 mL). The reaction vessel along with another identical vessel was closed and fixed on the vibration arms of a Retsch MM400 mixer mill, and was vibrated at a rate of 1800 rounds per minute (30 Hz) at room temperature for 30 min. After completion of the reaction, the resulting mixtures from the two runs were combined and extracted with dichloromethane and water. The organic layer was decanted, and the aqueous layer was extracted by dichloromethane (2 × 20 mL). The combined organic extracts were evaporated to remove the solvent in vacuo. The residue was separated by flash column chromatography on silica gel with ethyl acetate/petroleum ether as the eluent to afford products **2a**–**r**, **3s** *and* **3t**.

*3,4-Di-p-tolyl-1,2,5-oxadiazole 2-oxide* (**2a**). By following the general procedure, the reaction of **1a** (54.8 mg, 0.4 mmol) with NaCl (24.6 mg, 0.4 mmol), Oxone (246.7 mg, 0.4 mmol) and NEt_3_ (56 μL, 0.4 mmol) afforded **2a** (42.6 mg, 79% yield). White solid; ^1^H NMR (500 MHz, CDCl_3_) *δ* 7.42 (d, *J* = 8.1 Hz, 2H), 7.40 (d, *J* = 7.9 Hz, 2H), 7.24 (d, *J* = 7.9 Hz, 4H), 2.41 (s, 3H), 2.39 (s, 3H); ^13^C NMR (126 MHz, CDCl_3_) *δ* 156.4, 141.4, 141.0, 129.82 (2C), 129.78 (2C), 128.7 (2C), 128.3 (2C), 124.1, 120.2, 114.5, 20.66, 20.63; HRMS (FTMS-ESI) Calcd for C_16_H_15_N_2_O_2_ [M + H]^+^ 267.1128; found 267.1133.

*3,4-Diphenyl-1,2,5-oxadiazole 2-oxide* (**2b**). By following the general procedure, the reaction of **1b** (44 µL, 0.4 mmol) with NaCl (24.4 mg, 0.4 mmol), Oxone (247.4 mg, 0.4 mmol) and NEt_3_ (56 μL, 0.4 mmol) afforded **2b** (24.8 mg, 52% yield). White solid; ^1^H NMR (500 MHz, CDCl_3_) *δ* 7.56–7.49 (m, 5H), 7.48–7.40 (m, 5H); ^13^C NMR (126 MHz, CDCl_3_) *δ* 156.4, 131.1, 130.7, 129.2 (2C), 129.1 (2C), 128.9 (2C), 128.5 (2C), 126.8, 123.1, 114.4. The NMR data agreed with those in a literature report [11].

*3,4-Bis(4-methoxyphenyl)-1,2,5-oxadiazole 2-oxide* (**2c**). By following the general procedure, the reaction of **1c** (61.4 mg, 0.4 mmol) with NaCl (94.0 mg, 1.6 mmol), Oxone (249.6 mg, 0.4 mmol) and Na_2_CO_3_ (85.4 mg, 0.8 mmol) afforded **2c** (25.8 mg, 43% yield). White solid; ^1^H NMR (500 MHz, CDCl_3_) *δ* 7.47 (d, *J* = 8.9 Hz, 2H), 7.46 (d, *J* = 8.9 Hz, 2H), 6.95 (d, *J* = 8.9 Hz, 2H), 6.94 (d, *J* = 8.9 Hz, 2H), 3.85 (s, 3H), 3.84 (s, 3H); ^13^C NMR (126 MHz, CDCl_3_) *δ* 161.7, 161.1, 156.1, 130.4 (2C), 129.9 (2C), 119.1, 115.0, 114.6 (4C), 114.3, 55.52, 55.51. The NMR data agreed with those in a literature report [53].

*3,4-Bis(4-fluorophenyl)-1,2,5-oxadiazole 2-oxide* (**2d**). By following the general procedure, the reaction of **1d** (56.6 mg, 0.4 mmol) with NaCl (25.8 mg, 0.4 mmol), Oxone (248.3 mg, 0.4 mmol) and NEt_3_ (56 μL, 0.4 mmol) afforded **2d** (38.6 mg, 69% yield). White solid; ^1^H NMR (400 MHz, DMSO-*d*_6_) *δ* 7.62–7.52 (m, 4H), 7.42–7.35 (m, 4H); ^13^C NMR (101 MHz, DMSO-*d*_6_) *δ* 163.6 (d, *J*_C–F_ = 249.1 Hz), 163.0 (d, *J*_C–F_ = 249.2 Hz), 155.9, 131.6 (d, *J*_C–F_ = 8.9 Hz, 2C), 130.9 (d, *J*_C–F_ = 9.0 Hz, 2C), 122.7 (d, *J*_C–F_ = 3.3 Hz), 119.0 (d, *J*_C–F_ = 3.3 Hz), 116.4 (d, *J*_C–F_ = 22.2 Hz, 2C), 116.3 (d, *J*_C–F_ = 22.3 Hz, 2C), 114.2; ^19^F NMR (376 MHz, DMSO-*d*_6_) *δ* –108.83 to –108.98 (m, 2F); HRMS (FTMS-ESI) Calcd for C_14_H_8_F_2_N_2_O_2_Na [M + Na]^+^ 297.0446; found 297.0454.

*3,4-Bis(4-chlorophenyl)-1,2,5-oxadiazole 2-oxide* (**2e**). By following the general procedure, the reaction of **1e** (63.0 mg, 0.4 mmol) with NaCl (23.6 mg, 0.4 mmol), Oxone (248.1 mg, 0.4 mmol) and NEt_3_ (56 μL, 0.4 mmol) afforded **2e** (47.8 mg, 77% yield). White solid; ^1^H NMR (400 MHz, CDCl_3_) *δ* 7.49–7.38 (m, 8H); ^13^C NMR (126 MHz, CDCl_3_) *δ* 155.2, 137.7, 137.1, 130.0 (2C), 129.72 (2C), 129.70 (2C), 129.6 (2C), 125.0, 121.2, 113.5. The NMR data agreed with those in a literature report [54].

*3,4-Bis(4-bromophenyl)-1,2,5-oxadiazole 2-oxide* (**2f**). By following the general procedure, the reaction of **1f** (81.0 mg, 0.4 mmol) with NaCl (24.6 mg, 0.4 mmol), Oxone (247.6 mg, 0.4 mmol) and NEt_3_ (56 μL, 0.4 mmol) afforded **2f** (62.5 mg, 78% yield). White solid; ^1^H NMR (500 MHz, CDCl_3_) *δ* 7.58 (d, *J* = 8.6 Hz, 4H), 7.38 (d, *J* = 8.6 Hz, 4H); ^13^C NMR (126 MHz, CDCl_3_) *δ* 155.2, 137.8, 137.1, 130.0 (2C), 129.73 (2C), 129.71 (2C), 129.66 (2C), 125.0, 121.2, 113.5. The NMR data agreed with those in a literature report [55].

*3,4-Bis(4-(methoxycarbonyl)phenyl)-1,2,5-oxadiazole 2-oxide* (**2g**). By following the general procedure and prolonging the reaction time to 60 min, the reaction of **1g** (72.8 mg, 0.4 mmol) with NaCl (25.2 mg, 0.4 mmol), Oxone (247.5 mg, 0.4 mmol) and NEt_3_ (56 μL, 0.4 mmol) afforded **2g** (44.6 mg, 62% yield). White solid; ^1^H NMR (500 MHz, CDCl_3_) *δ* 8.13 (d, *J* = 8.4 Hz, 2H), 8.11 (d, *J* = 8.5 Hz, 2H), 7.60 (d, *J* = 8.3 Hz, 4H), 3.96 (s, 3H), 3.95 (s, 3H); ^13^C NMR (126 MHz, CDCl_3_) *δ* 166.1, 166.0, 155.4, 132.8, 132.2, 130.6, 130.5 (2C), 130.3 (2C), 128.7 (2C), 128.5 (2C), 127.0, 113.6, 52.69, 52.67; HRMS (FTMS-ESI) Calcd for C_18_H_15_N_2_O_6_ [M + H]^+^ 355.0925; found 355.0930.

*3,4-Bis(4-nitrophenyl)-1,2,5-oxadiazole 2-oxide* (**2h**). By following the general procedure, the reaction of **1h** (67.8 mg, 0.4 mmol) with NaCl (26.2 mg, 0.4 mmol), Oxone (248.0 mg, 0.4 mmol) and NEt_3_ (56 μL, 0.4 mmol) afforded **2h** (20.1 mg, 30% yield). When NEt_3_ is replaced by NaO*^t^*Bu, a much better result was obtained. Thus, the reaction of **1h** (68.0 mg, 0.4 mmol) with NaCl (47.6 mg, 0.8 mmol), Oxone (739.6 mg, 1.2 mmol) and NaO*^t^*Bu (39.0 mg, 0.4 mmol) afforded **2h** (61.2 mg, 91% yield). White solid; ^1^H NMR (400 MHz, CD_2_Cl_2_) *δ* 8.34 (d, *J* = 8.7 Hz, 2H), 8.32 (d, *J* = 8.7 Hz, 2H), 7.73 (d, *J* = 8.7 Hz, 2H), 7.72 (d, *J* = 8.7 Hz, 2H); ^13^C NMR (101 MHz, CD_2_Cl_2_) *δ* 153.7, 148.8, 148.1, 131.2, 128.9 (2C), 128.8 (2C), 127.8, 123.8 (2C), 123.6 (2C), 112.0. The NMR data agreed with those in a literature report [56].

*3,4-Di-m-tolyl-1,2,5-oxadiazole 2-oxide* (**2i**). By following the general procedure, the reaction of **1i** (56.4 mg, 0.4 mmol) with NaCl (25.7 mg, 0.4 mmol), Oxone (247.2 mg, 0.4 mmol) and NEt_3_ (56 μL, 0.4 mmol) afforded **2i** (41.5 mg, 75% yield). White solid; ^1^H NMR (500 MHz, DMSO-*d*_6_) *δ* 7.42–7.32 (m, 6H), 7.26–7.19 (m, 2H), 2.31 (s, 3H), 2.30 (s, 3H); ^13^C NMR (126 MHz, DMSO-*d*_6_) *δ* 156.7, 138.5, 138.3, 131.7, 131.4, 129.1, 128.9, 128.8, 128.5, 126.2, 126.1, 125.4, 122.6, 114.6, 20.90, 20.86; HRMS (FTMS-ESI) Calcd for C_16_H_15_N_2_O_2_ [M + H]^+^ 267.1128; found 267.1138.

*3,4-Bis(3-fluorophenyl)-1,2,5-oxadiazole 2-oxide* (**2j**). By following the general procedure, the reaction of **1j** (56.5 mg, 0.4 mmol) with NaCl (24.5 mg, 0.4 mmol), Oxone (248.0 mg, 0.4 mmol) and NEt_3_ (56 μL, 0.4 mmol) afforded **2j** (38.5 mg, 69% yield). White solid; ^1^H NMR (500 MHz, DMSO-*d*_6_) *δ* 7.62–7.55 (m, 2H), 7.50–7.36 (m, 4H), 7.36–7.29 (m, 2H); ^13^C NMR (126 MHz, DMSO-*d*_6_) *δ* 161.9 (d, *J*_C–F_ = 245.5 Hz), 161.8 (d, *J*_C–F_ = 244.9 Hz), 155.6 (d, *J*_C–F_ = 2.8 Hz), 131.5 (d, *J*_C–F_ = 8.6 Hz), 131.3 (d, *J*_C–F_ = 8.6 Hz), 128.2 (d, *J*_C–F_ = 8.7 Hz), 125.4 (d, *J*_C–F_ = 3.1 Hz), 124.7 (d, *J*_C–F_ = 3.2 Hz), 124.6 (d, *J*_C–F_ = 9.1 Hz), 118.2 (d, *J*_C–F_ = 21.0 Hz), 117.9 (d, *J*_C–F_ = 21.1 Hz), 115.8 (d, *J*_C–F_ = 24.2 Hz), 115.3 (d, *J*_C–F_ = 23.8 Hz), 114.0 (d, *J*_C–F_ = 2.7 Hz); ^19^F NMR (471 MHz, DMSO-*d*_6_) *δ* –111.42 to –111.50 (m, 2F); HRMS (FTMS-ESI) Calcd for C_14_H_8_F_2_N_2_O_2_Na [M + Na]^+^ 297.0446; found 297.0456.

*3,4-Bis(3-chlorophenyl)-1,2,5-oxadiazole 2-oxide* (**2k**). By following the general procedure, the reaction of **1k** (63.4 mg, 0.4 mmol) with NaCl (24.0 mg, 0.4 mmol), Oxone (249.4 mg, 0.4 mmol) and NEt_3_ (56 μL, 0.4 mmol) afforded **2k** (54.6 mg, 87% yield). White solid; ^1^H NMR (500 MHz, DMSO-*d*_6_) *δ* 7.68 (ddd, *J* = 8.1, 2.3, 1.1 Hz, 1H), 7.66–7.61 (m, 2H), 7.59 (t, *J* = 1.8 Hz, 1H), 7.59–7.53 (m, 2H), 7.45 (dd, *J* = 7.7, 1.4 Hz, 1H), 7.43 (dd, *J* = 7.9, 1.4 Hz, 1H); ^13^C NMR (126 MHz, DMSO-*d*_6_) *δ* 155.5, 133.7, 133.5, 131.16, 131.15, 130.9, 130.8, 128.6, 128.1, 128.0, 127.8, 127.2, 124.6, 113.9; HRMS (FTMS-ESI) Calcd for C_14_H_9_^35^Cl_2_N_2_O_2_ [M + H]^+^ 307.0036; found 307.0031.

*3,4-Bis(3-bromophenyl)-1,2,5-oxadiazole 2-oxide* (**2l**). By following the general procedure, the reaction of **1l** (81.1 mg, 0.4 mmol) with NaCl (25.5 mg, 0.4 mmol), Oxone (248.1 mg, 0.4 mmol) and NEt_3_ (56 μL, 0.4 mmol) afforded **2l** (72.8 mg, 91% yield). White solid; ^1^H NMR (400 MHz, DMSO-*d*_6_) *δ* 7.84–7.79 (m, 1H), 7.79–7.74 (m, 2H), 7.74–7.70 (m, 1H), 7.52–7.46 (m, 4H); ^13^C NMR (101 MHz, DMSO-*d*_6_) *δ* 155.4, 134.0, 133.6, 131.4, 131.3, 131.1, 130.9, 128.3, 128.1, 127.5, 124.8, 122.0, 121.8, 113.8; HRMS (FTMS-ESI) Calcd for C_14_H_9_^79^Br_2_N_2_O_2_ [M + H]^+^ 394.9025; found 394.9025.

*3,4-Bis(3,4-dimethylphenyl)-1,2,5-oxadiazole 2-oxide* (**2m**). By following the general procedure, the reaction of **1m** (61.2 mg, 0.4 mmol) with NaCl (24.6 mg, 0.4 mmol), Oxone (247.9 mg, 0.4 mmol) and NEt_3_ (56 μL, 0.4 mmol) afforded **2m** (51.6 mg, 85% yield). White solid; ^1^H NMR (400 MHz, DMSO-*d*_6_) *δ* 7.38 (d, *J* = 1.9 Hz, 1H), 7.37 (d, *J* = 1.9 Hz, 1H), 7.25 (d, *J* = 8.0 Hz, 1H), 7.24 (d, *J* = 7.9 Hz, 1H), 7.14 (dd, *J* = 7.9, 1.9 Hz, 1H), 7.12 (dd, *J* = 8.0, 1.9 Hz, 1H), 2.272 (s, 3H), 2.267 (s, 3H), 2.23 (s, 3H), 2.22 (s, 3H); ^13^C NMR (101 MHz, DMSO-*d*_6_) *δ* 156.6, 139.8, 139.4, 137.3, 137.1, 129.94, 129.87, 129.4, 128.8, 126.4, 125.7, 123.8, 120.0, 114.5, 19.40, 19.36 (2C), 19.3; HRMS (FTMS-ESI) Calcd for C_18_H_18_N_2_O_2_Na [M + Na]^+^ 317.1260; found 317.1257.

*3,4-Bis(3,5-dimethylphenyl)-1,2,5-oxadiazole 2-oxide* (**2n**). By following the general procedure, the reaction of **1n** (60.6 mg, 0.4 mmol) with NaCl (24.4 mg, 0.4 mmol), Oxone (247.8 mg, 0.4 mmol) and NEt_3_ (56 μL, 0.4 mmol) afforded **2n** (55.1 mg, 92% yield). White solid; ^1^H NMR (400 MHz, DMSO-*d*_6_) *δ* 7.21 (s, 1H), 7.17 (s, 1H), 7.13–7.09 (m, 4H), 2.25 (s, 6H), 2.24 (s, 6H); ^13^C NMR (101 MHz, DMSO-*d*_6_) *δ* 156.6, 138.3 (2C), 138.1 (2C), 132.5, 132.1, 126.4 (2C), 126.2, 125.8 (2C), 122.5, 114.4, 20.8 (2C), 20.7 (2C); HRMS (FTMS-ESI) Calcd for C_18_H_19_N_2_O_2_ [M + H]^+^ 295.1441; found 295.1427.

*3,4-Bis(4-fluoro-3-methylphenyl)-1,2,5-oxadiazole 2-oxide* (**2o**). By following the general procedure, the reaction of **1o** (62.8 mg, 0.4 mmol) with NaCl (24.8 mg, 0.4 mmol), Oxone (247.4 mg, 0.4 mmol) and NEt_3_ (56 μL, 0.4 mmol) afforded **2o** (54.8 mg, 88% yield). White solid; ^1^H NMR (500 MHz, DMSO-*d*_6_) *δ* 7.57–7.49 (m, 2H), 7.33–7.26 (m, 4H), 2.25 (d, *J* = 2.0 Hz, 3H), 2.24 (d, *J* = 2.0 Hz, 3H); ^13^C NMR (126 MHz, DMSO-*d*_6_) *δ* 162.1 (d, *J*_C–F_ = 248.5 Hz), 161.6 (d, *J*_C–F_ = 248.6 Hz), 155.9, 132.3 (d, *J*_C–F_ = 5.7 Hz), 131.6 (d, *J*_C–F_ = 5.9 Hz), 129.1 (d, *J*_C–F_ = 9.0 Hz), 128.2 (d, *J*_C–F_ = 8.9 Hz), 125.6 (d, *J*_C–F_ = 20.5 Hz), 125.5 (d, *J*_C–F_ = 20.3 Hz), 122.4 (d, *J*_C–F_ = 3.6 Hz), 118.7 (d, *J*_C–F_ = 3.4 Hz) 115.9 (d, *J*_C–F_ = 23.0 Hz, 2C), 114.1, 14.16 (d, *J*_C–F_ = 3.2 Hz), 14.08 (d, *J*_C–F_ = 3.1 Hz); ^19^F NMR (471 MHz, DMSO-*d*_6_) *δ* –113.08 to –113.26 (m, 2F); HRMS (FTMS-ESI) Calcd for C_16_H_12_F_2_N_2_O_2_Na [M + Na]^+^ 325.0759; found 325.0752.

*3,4-Dibenzyl-1,2,5-oxadiazole 2-oxide* (**2p**). By following the general procedure, the reaction of **1p** (55.6 mg, 0.4 mmol) with NaCl (48.0 mg, 0.8 mmol), Oxone (251.8 mg, 0.4 mmol) and Na_2_CO_3_ (85.0 mg, 0.8 mmol) afforded **2p** (38.4 mg, 70% yield). White solid; ^1^H NMR (500 MHz, CDCl_3_) *δ* 7.33–7.26 (m, 3H), 7.25–7.21 (m, 3H), 7.13–7.08 (m, 2H), 7.00–6.95 (m, 2H), 3.86 (s, 2H), 3.61 (s, 2H). ^13^C NMR (126 MHz, CDCl_3_) *δ* 156.8, 134.0, 133.8, 129.1 (2C), 129.0 (2C), 128.8 (2C), 128.4 (2C), 127.72, 127.70, 115.4, 32.1, 28.4; HRMS (FTMS-ESI) Calcd for C_16_H_15_N_2_O_2_ [M + Na]^+^ 267.1128; found 267.1125.

*3,4-Diphenethyl-1,2,5-oxadiazole 2-oxide* (**2q**). By following the general procedure, the reaction of **1q** (61.0 mg, 0.4 mmol) with NaCl (47.6 mg, 0.8 mmol), Oxone (246.2 mg, 0.4 mmol) and Na_2_CO_3_ (85.8 mg, 0.8 mmol) afforded **2q** (47.2 mg, 78% yield). White solid; ^1^H NMR (500 MHz, CD_2_Cl_2_) *δ* 7.28 (t, *J* = 7.2 Hz, 4H), 7.22 (t, *J* = 7.0 Hz, 2H), 7.10 (d, *J* = 7.1 Hz, 4H), 2.85 (t, *J* = 7.3 Hz, 2H), 2.82 (t, *J* = 7.9 Hz, 2H), 2.64 (t, *J* = 7.2 Hz, 2H), 2.49 (t, *J* = 7.9 Hz, 2H); ^13^C NMR (126 MHz, CDCl_3_) *δ* 157.7, 139.7, 139.4, 129.0 (2C), 128.8 (2C), 128.5 (2C), 128.4 (2C), 127.0, 126.8, 115.5, 32.7, 30.8, 27.2, 24.7; HRMS (FTMS-ESI) Calcd for C_18_H_19_N_2_O_2_ [M + H]^+^ 295.1441; found 295.1444.

*3,4-Dipentyl-1,2,5-oxadiazole 2-oxide* (**2r**). By following the general procedure, the reaction of **1r** (48.4 mg, 0.4 mmol) with NaCl (47.6 mg, 0.8 mmol), Oxone (252.0 mg, 0.4 mmol) and Na_2_CO_3_ (127.2 mg, 1.2 mmol) afforded **2r** (23.6 mg, 50% yield). Colourless liquid; ^1^H NMR (500 MHz, CDCl_3_) *δ* 2.62 (t, *J* = 7.7 Hz, 1H), 2.50 (t, *J* = 7.7 Hz, 1H), 1.79–1.69 (m, 2H), 1.67–1.57 (m, 3H), 1.41–1.27 (m, 9H), 0.95–0.86 (m, 6H). ^13^C NMR (126 MHz, CDCl_3_) *δ* 158.1, 116.2, 31.4, 31.3, 26.5, 25.8, 25.2, 22.5, 22.38, 22.35, 14.00, 13.98; HRMS (FTMS-ESI) Calcd for C_12_H_22_N_2_O_2_Na [M + Na]^+^ 249.1573; found 249.1570.

*2-Methylbenzonitrile nitrile oxide* (**3s**). By following the general procedure, the reaction of **1s** (55.4 mg, 0.4 mmol) with NaCl (24.4 mg, 0.4 mmol), Oxone (249.6 mg, 0.4 mmol) and NEt_3_ (56 μL, 0.4 mmol) afforded **3s** (46.9 mg, 86% yield). White solid; ^1^H NMR (400 MHz, CDCl_3_) *δ* 7.46 (d, *J* = 7.8 Hz, 1H), 7.38 (t, *J* = 7.6 Hz, 1H), 7.28 (d, *J* = 7.2 Hz, 1H), 7.23 (t, *J* = 7.6 Hz, 1H); ^13^C NMR (101 MHz, CDCl_3_) *δ* 141.7, 132.6, 131.0, 130.4, 126.5, 113.9, 20.8; HRMS (FTMS-ESI) Calcd for C_8_H_7_NONa [M + Na]^+^ 156.0420; found 156.0425.

*2,4,6-Trimethylbenzonitrile nitrile oxide* (**3t**). By following the general procedure, the reaction of **1t** (66.4 mg, 0.4 mmol) with NaCl (24.4 mg, 0.4 mmol), Oxone (369.2 mg, 0.6 mmol) and Na_2_CO_3_ (86.6 mg, 0.8 mmol) afforded **3t** (59.0 mg, 90% yield). White solid; ^1^H NMR (400 MHz, DMSO-*d*_6_) *δ* 7.03 (s, 2H), 2.36 (s, 6H), 2.27 (s, 3H); ^13^C NMR (101 MHz, DMSO-*d*_6_) *δ* 141.4, 140.9, 128.2, 110.3, 21.0, 20.2. The NMR data agreed with those in a literature report [41].

### 3.3. Mechanochemial Synthesis of ***2a*** from ***1a*** and ***1a’***

A mixture of *Z*-isomer **1a’** and *E*-isomer **1a** (7:1) was used to replace **1a**. By following the same procedure for the mechanochemical synthesis of **2a** from **1a**, the reaction of isomeric **1a’** and **1a** (7:1) (54.6 mg, 0.4 mmol) with NaCl (24.2 mg, 0.4 mmol), Oxone (247.2 mg, 0.4 mmol) and NEt_3_ (56 μL, 0.4 mmol) afforded **2a** (40.4 mg, 75% yield).

### 3.4. Mechanochemial Synthesis of ***4a*** from ***1a*** and ***2a*** from ***4a***

A mixture of **1a** (27.3 mg, 0.2 mmol), Oxone (124.4 mg, 0.2 mmol) and NaCl (12.3 mg, 0.2 mmol) together with four stainless-steel balls (5 mm in diameter) was introduced into a stainless-steel jar (5 mL). The reaction vessel and another same vessel were closed and fixed on the vibration arms of a Retsch MM400 mixer mill and were vibrated at a rate of 1800 rounds per minute (30 Hz) at room temperature for 30 min. After completion of the reaction, the resulting mixtures from the two runs were combined and extracted with dichloromethane and water. The organic layer was decanted, and the aqueous layer was extracted by dichloromethane (2 × 20 mL). The combined organic extracts were evaporated to remove the solvent in vacuo. The residue was separated by flash column chromatography on silica gel with ethyl acetate/petroleum ether as the eluent to afford **4a** (59.8 mg, 87% yield). CH_3_CN, the best LAG in the overall dimerization reaction, was also examined for the synthesis of **4a**. By following the above procedure, the reaction of **1a** (56.8 mg, 0.4 mmol) with NaCl (23.8 mg, 0.4 mmol) and Oxone (245.8 mg, 0.4 mmol) in the presence of CH_3_CN (44 µL) afforded **4a** (49.0 mg, 69% yield).

A mixture of **4a** (34.2 mg, 0.2 mmol) and NEt_3_ (28 μL, 0.2 mmol) together with four stainless-steel balls (5 mm in diameter) was introduced into a stainless-steel jar (5 mL). The reaction vessel and another same vessel were closed and fixed on the vibration arms of a Retsch MM400 mixer mill and were vibrated at a rate of 1800 rounds per minute (30 Hz) at room temperature for 30 min. After completion of the reaction, the reaction vessels were washed with ethyl acetate three times (3 × 5 mL) and the combined solution was evaporated to remove the solvent in vacuo. The residue was separated by flash column chromatography on silica gel with ethyl acetate/petroleum ether as the eluent to afford **2a** (41.2 mg, 77% yield). CH_3_CN, the best LAG in the overall dimerization reaction, was also examined for the synthesis of **2a** from **4a**. By following the above procedure, the reaction of **4a** (68.2 mg, 0.4 mmol) with NEt_3_ (56 μL, 0.4 mmol) in the presence of CH_3_CN (12 µL) afforded **2a** (39.6 mg, 74% yield).

### 3.5. Deoxygenation Reaction of ***2a***

A mixture of **2a** (27.0 mg, 0.1 mmol) and triethyl phosphite (0.5 mL) was heated at 165 °C under an argon atmosphere for 12 h. After completion of the reaction, the reaction vessels were washed with ethyl acetate three times (3 × 5 mL), and the combined solution was evaporated to remove the solvent in vacuo. The residue was separated by flash column chromatography on silica gel with ethyl acetate/petroleum ether as the eluent to afford **5a** (23.1 mg, 91% yield). ^1^H NMR (500 MHz, CDCl_3_) *δ* 7.43 (d, *J* = 8.0 Hz, 4H), 7.23 (d, *J* = 8.0 Hz, 4H), 2.41 (s, 6H); ^13^C NMR (101 MHz, CDCl_3_) *δ* 153.2 (2C), 140.8 (2C), 129.7 (4C), 128.9 (4C), 123.1 (2C), 21.6 (2C); HRMS (FTMS-ESI) Calcd for C_16_H_15_N_2_O [M + H]^+^ 251.1179; found 251.1175.

### 3.6. Synthesis of ***2a*** in Liquid Phase

To a stirred solution of **1a** (27.5 mg, 0.2 mmol) in CH_3_CN (2 mL) were added NaCl (12.1 mg, 0.2 mmol), Oxone (123.4 mg, 0.2 mmol) and NEt_3_ (28 μL, 0.2 mmol). The reaction mixture was allowed to stir at room temperature for 2 h. Then, the reaction mixture was filtered through a silica gel plug with ethyl acetate as the eluent and, subsequently, the solvent was removed under reduced pressure. The residue was purified by flash chromatography on silica gel with petroleum ether/ethyl acetate as eluent to give product **2a** (12.7 mg, 47% yield).

## 4. Conclusions

In conclusion, we have successfully developed a solvent-free dimerization reaction of aldoximes to obtain furoxans in the presence of sodium chloride, Oxone and a base under solvent-free ball-milling conditions. The starting materials are easily available, and various aromatic and aliphatic aldoximes can be employed as the substrates. This protocol has advantages of ambient reaction conditions, high yields, solvent-free and catalyst-free conditions. Finally, a plausible reaction mechanism is proposed to explain the formation of furoxans.

## Figures and Tables

**Table 1:**
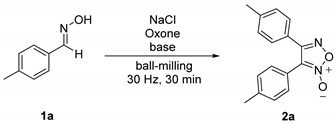
Optimization of the reaction conditions ^a^.

Entry	NaCl (Equiv.)	Oxone (Equiv.)	Base (Equiv.)	Yield of 2a (%) ^b^
1	1.0	1.0	Na_2_CO_3_ (1.0)	8
2	1.0	1.0	NaO*^t^*Bu (1.0)	trace
3	1.0	1.0	NaOAc (1.0)	trace
4	1.0	1.0	NaHCO_3_ (1.0)	trace
5	1.0	1.0	K_2_CO_3_ (1.0)	36
6	1.0	1.0	Cs_2_CO_3_ (1.0)	7
7	1.0	1.0	DMAP (1.0)	0
8	1.0	1.0	DBU (1.0)	0
9	1.0	1.0	DABCO (1.0)	0
**10**	**1.0**	**1.0**	**NEt_3_** **(1.0)**	**79**
11	1.0	1.0	NEt_3_ (1.25)	69
12	1.0	1.0	NEt_3_ (1.5)	53
13	1.0	1.0	NEt_3_ (0.75)	62
14	1.0	1.0	NEt_3_ (0.5)	38
15 ^c^	1.0	1.0	NEt_3_ (1.0)	trace
16 ^d^	1.0	1.0	NEt_3_ (1.0)	47
17 ^e^	1.0	1.0	NEt_3_ (1.0)	78
18	1.5	1.0	NEt_3_ (1.0)	79
19	1.0	1.5	NEt_3_ (1.0)	77
20 ^f,g^	1.0	1.0	NEt_3_ (1.0)	50
21 ^f,h^	1.0	1.0	NEt_3_ (1.0)	55
22 ^f,i^	1.0	1.0	NEt_3_ (1.0)	51
23 ^f,j^	1.0	1.0	NEt_3_ (1.0)	71

^a^ Unless otherwise stated, the reactions were performed in a stainless-steel jar (5 mL) with **1a** (0.2 mmol), NaCl (1.0 equiv.), Oxone (1.0 equiv.) and base (1.0 equiv.) together with four stainless-steel balls (5 mm in diameter) using a Retsch MM400 mixer mill at 30 Hz for 30 min. ^b^ Isolated yield based on **1a**. ^c^ Magnetic stirring for 2 h instead of ball milling. ^d^ Reaction time was 15 min. ^e^ Reaction time was 40 min. ^f^ A liquid (22 µL, *η* = 0.17 µL/mg) was added as a LAG agent. ^g^ EtOH was added. ^h^ DCM was added. ^i^ EtOAc was added. ^j^ CH_3_CN was added.

## Data Availability

Not applicable.

## Supplementary Materials

The following are available online at www.mdpi.com/article/10.3390/molecules27082604/s1, Calculation of green chemistry metrics for **2a**, NMR spectra of **1a** & **1a’**, **2a**–**r**, **3s**, **3t** and **5a**; X-ray structure and crystal data of **2****c**. References [57,58,59,60,61,62] are cited in the Supplementary Materials.

## Sample Availability

Samples of the compounds are available from the authors.
