# Supplementary material for: Mechanochemical Dimerization of Aldoximes to Furoxans
Source: Molecules. 2022 Apr 18;27(8):2604. doi: 10.3390/molecules27082604 (PMC9027020; doi:10.3390/molecules27082604)
Supplement: Supplementary file 1 [file molecules-27-02604-s001.zip › molecules-1657219-supplementary.pdf]

Supplementary Materials

Mechanochemical Dimerization of Aldoximes to Furoxans

Run-Kai Fang [1], Kuan Chen [1], Chuang Niu [1] and Guan-Wu Wang [1,2,*]

[1]Hefei National Laboratory for Physical Sciences at Microscale and Department of Chemistry, University of Science and Technology of China, Hefei 230026, China
[2]State Key Laboratory of Applied Organic Chemistry, Lanzhou University, Lanzhou 730000, China

[*]Correspondence: gwang@ustc.edu.cn; Tel: +86-551-6360-7864

Table of Contents:

1. Calculation of green chemistry metrics for the synthesis of **2a** S2–3
2. NMR spectra of compounds **1a** & **1a'**, **2a-r**, **3s**, **3t** and **5a** S4–27
3. X-Ray single-crystal data of compound **2c** S27–28
4. References S29

1. Calculation of green chemistry metrics for the synthesis of 2a.

Environmental factor (*E* factor) is a metric for quantifying the actual amount of waste produced in the process. The waste was originally defined as "everything but the desired product" [1–3]. According to the recently proposed new *E* factor derivatives, we calculated two *E* factors: the complete *E* factor (cEF); the simple *E* factor (sEF) [4]. The cEF metric accounts for all process materials, including raw materials, reagents, solvents water and product. The sEF metric discounts water and solvents.

$$\mathrm{cEF} = \frac{\sum \mathrm{m(raw\ materials)} + \sum \mathrm{m(reagents)} + \sum \mathrm{m(solvents)} + \sum \mathrm{m(water)} - \mathrm{m(product)}}{\mathrm{m(product)}}$$

$$\mathrm{sEF} = \frac{\sum \mathrm{m(raw\ materials)} + \sum \mathrm{m(reagents)} - \mathrm{m(product)}}{\mathrm{m(product)}}$$

Higher cEF and sEF mean more waste. The ideal value of cEF and sEF is zero.

Atom economy (AE) is defined as "how much of the reactants end up in the product" [5]. It can be calculated as the molecular weight of the desired product divided by the total molecular weights of all reactants produced in the stoichiometric equation.

$$\mathrm{AE} = \frac{\mathrm{MW(product)}}{\sum \mathrm{MW(raw\ materials)}} \times 100\%$$

The ideal value of AE is 100%.

Reaction mass efficiency (RME) takes into account yields, the actual molar quantities of reactants, and the concepts of atom efficiency. RME is defined as the percentage of the mass of the isolated product divided by the total mass of the reactants used in the reaction [6].

$$\mathrm{RME} = \frac{\mathrm{m(product)}}{\sum \mathrm{m(raw\ materials)}} \times 100\%$$

The ideal value of RME is 100%.

Calculation of green chemistry metrics for the mechanochemical synthesis of 2a

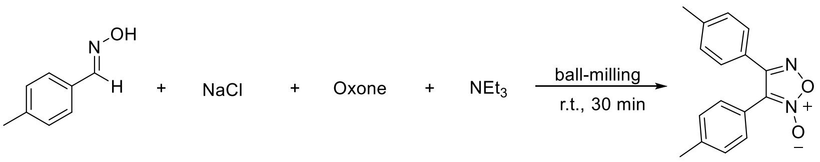

1a				2a
(MW: 135.17)	(MW: 58.44)	(MW: 614.78)	(MW: 101.19)	(MW: 266.30)
0.4 mmol	0.4 mmol	0.4 mmol	0.4 mmol	0.16 mmol
54.8 mg	24.6 mg	246.7 mg	40.8 mg	42.6 mg, 79%

dichloromethane: density 1.33 g/mL at 25 °C
water: density 1.00 g/mL at 25 °C
ethyl acetate: density 0.90 g/mL at 25 °C
petroleum ether: density 0.64 g/mL at 25 °C
workup: dichloromethane, 46 mL, 61.2 g; water, 6 mL, 6.0 g
purification: ethyl acetate, 8 mL, 7.2 g; petroleum ether, 440 mL, 281.6 g

$$cEF = \frac{54.8 + (24.6+246.7+40.8)+(61200+7200+281600)+6000 - 42.6}{42.6}$$

= 8364.4 kg waste/1 kg product

$$sEF = \frac{54.8 + (24.6+246.7+40.8) - 42.6}{42.6}$$

= 7.6 kg waste/1 kg product

$$AE = \frac{266.30}{135.17 \times 2} \times 100\% = 99\%$$

$$RME = \frac{42.6}{54.8} \times 100\% = 78\%$$

Calculation of green chemistry metrics for solution-based synthesis of 2a

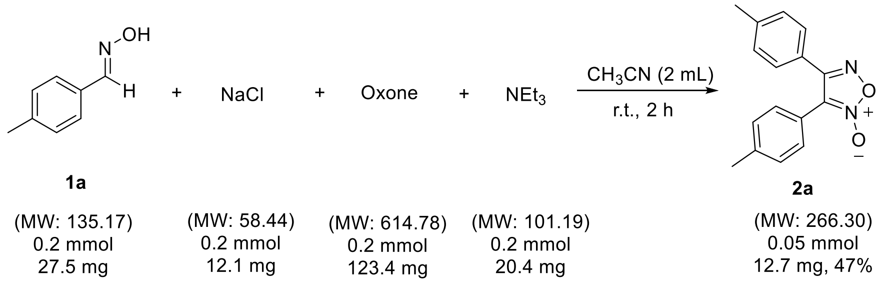

acetonitrile: density 0.79 g/mL at 25 °C
ethyl acetate: density 0.90 g/mL at 25 °C
petroleum ether: density 0.64 g/mL at 25 °C
workup: acetonitrile, 2 mL, 1.6 g; ethyl acetate, 15 mL, 13.5 g
purification: ethyl acetate, 6 mL, 5.4 g; petroleum ether, 270 mL, 172.8 g

$$cEF = \frac{27.5 + (12.1+123.4+20.4)+(1600+13500+5400+172800) - 12.7}{12.7}$$

= 15233.9 kg waste/1 kg product

$$sEF = \frac{27.5 + (12.1+123.4+20.4) - 12.7}{12.7}$$

= 13.4 kg waste/1 kg product

$$AE = \frac{266.30}{135.17 \times 2} \times 100\% = 99\%$$

$$RME = \frac{12.7}{27.5} \times 100\% = 46\%$$

2. NMR spectra of compounds 1a & 1a', 2a-r, 3s, 3t and 5a.

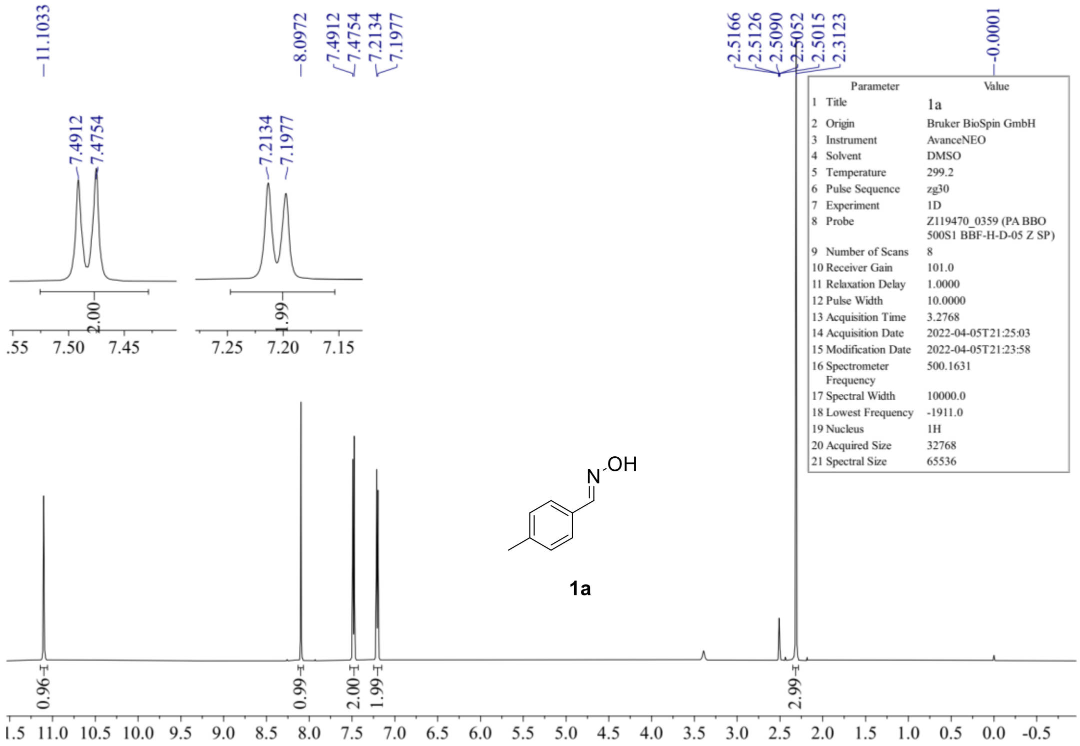

Figure S1. ^{1}H NMR (500 MHz, DMSO-d_6) of compound **1a**.

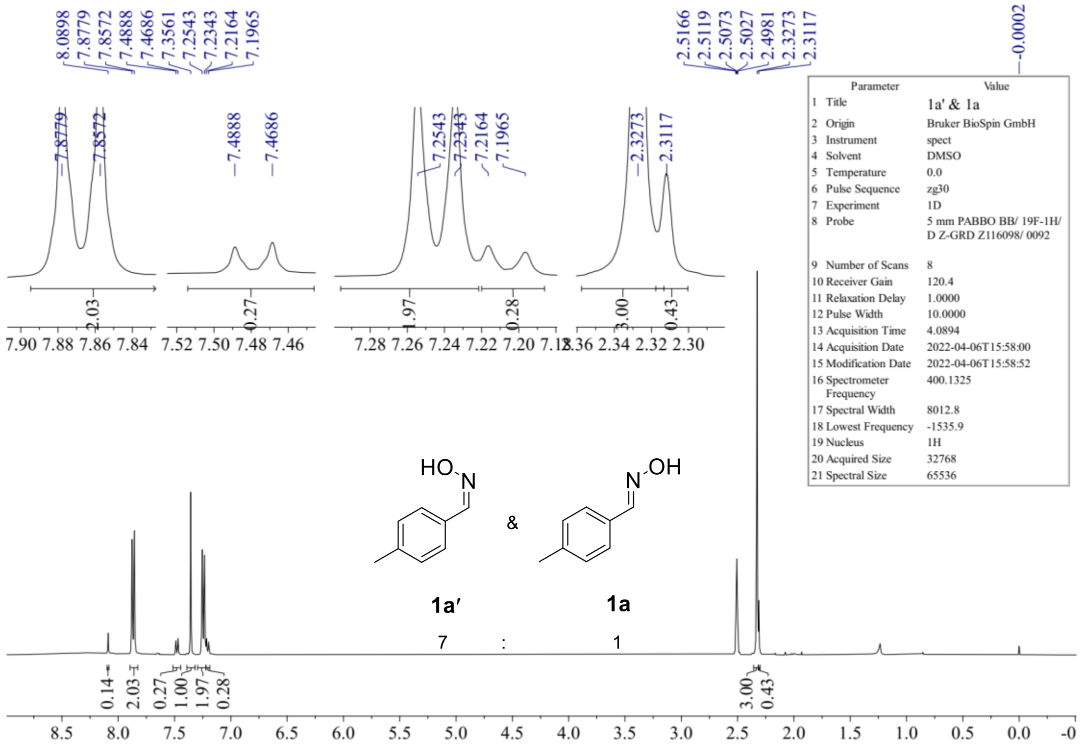

Figure S2. ^{1}H NMR (400 MHz, DMSO-d_6) of compound **1a** and **1a'**.

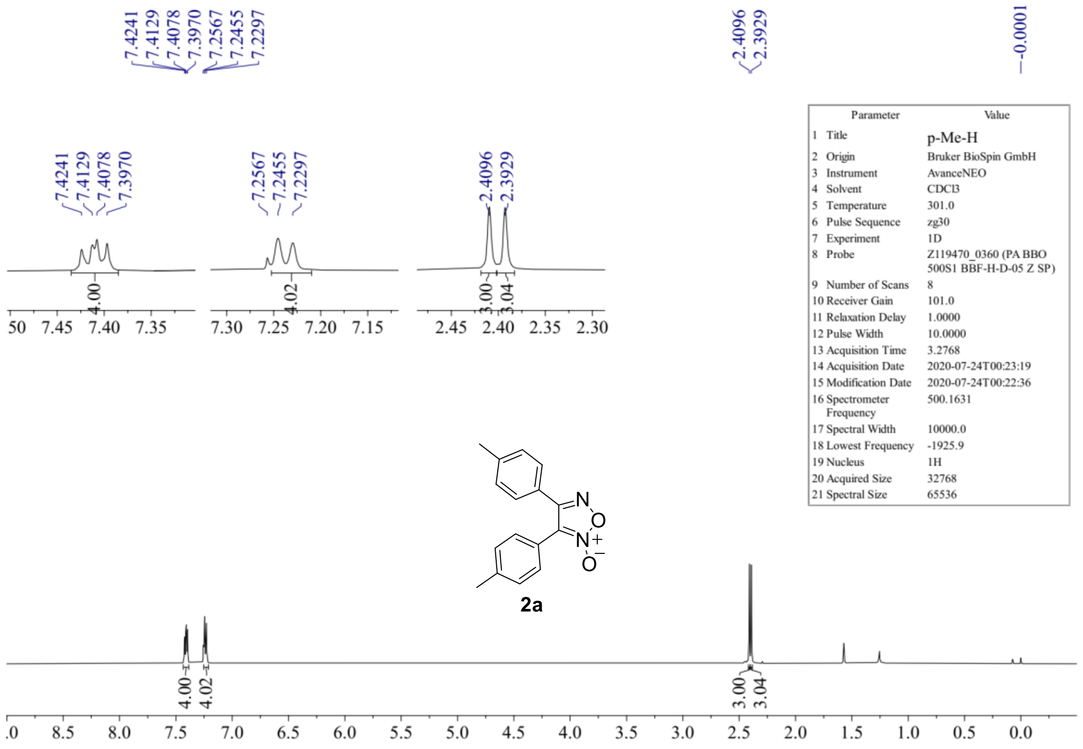

Figure S3. ^{1}H NMR (500 MHz, $CDCl_3$) of compound **2a**.

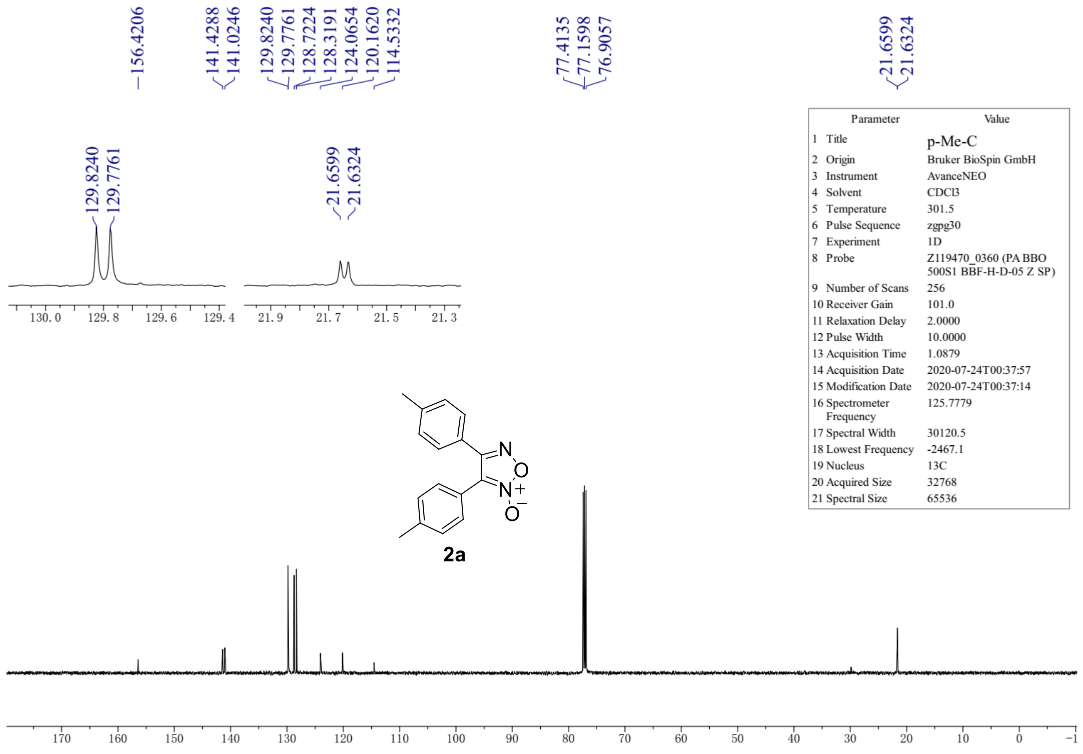

Figure S4. ^{13}C NMR (126 MHz, $CDCl_3$) of compound **2a**.

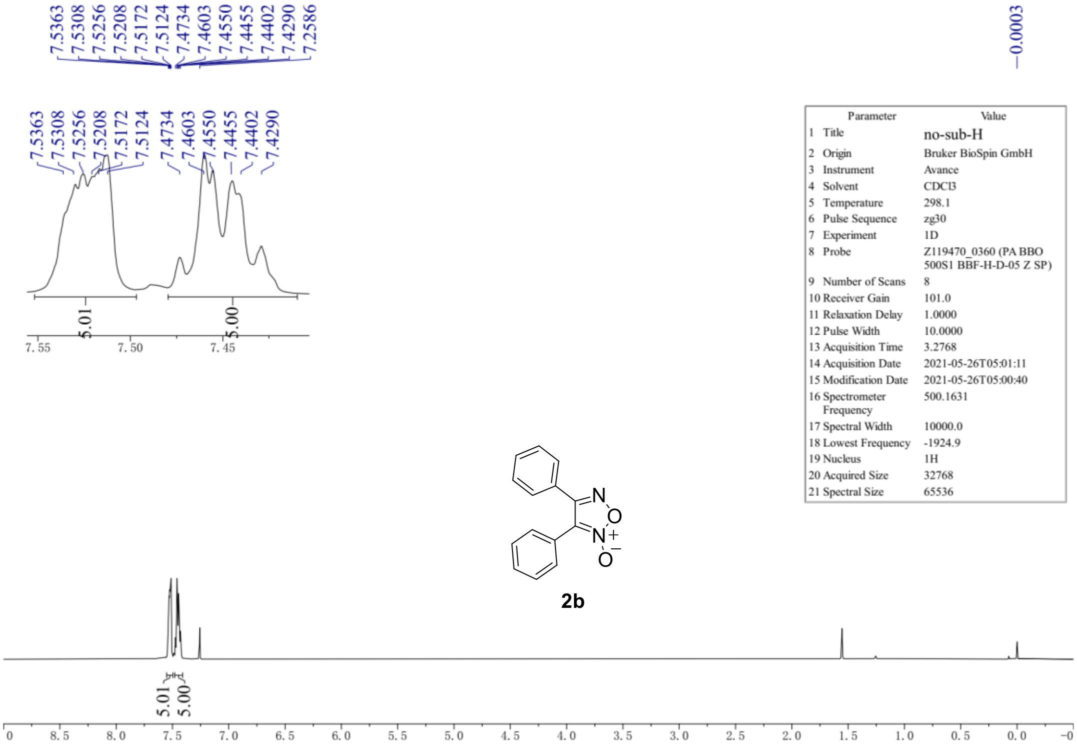

Figure S5. ^{1}H NMR (500 MHz, $CDCl_3$) of compound **2b**.

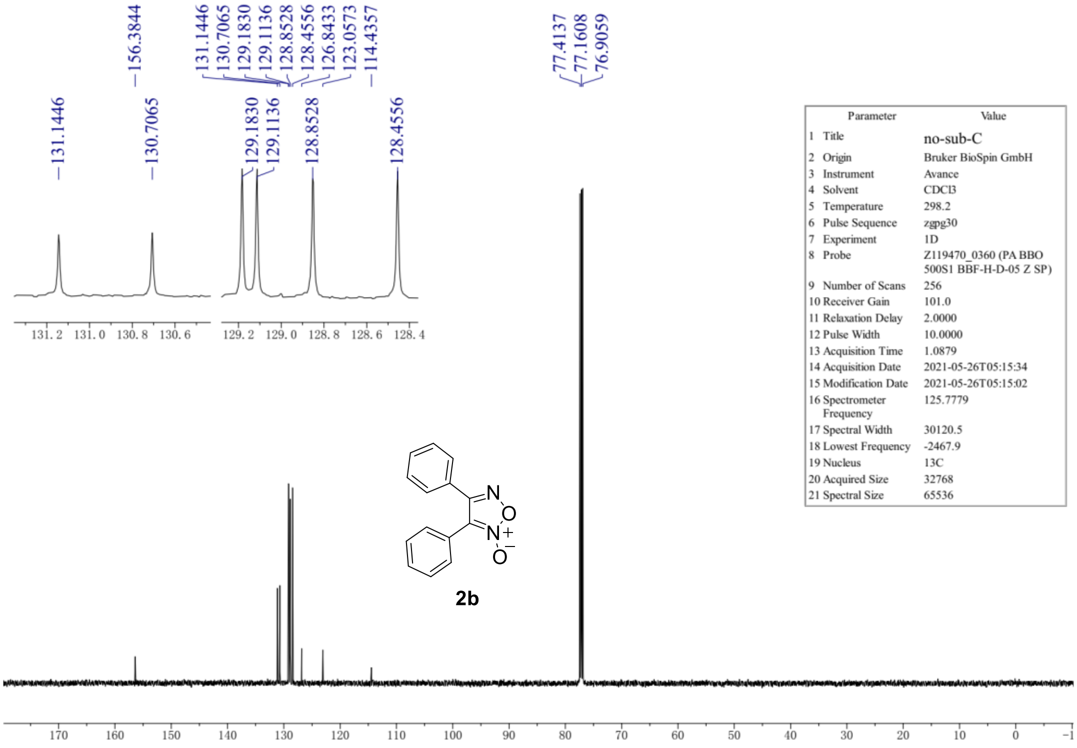

Figure S6. ^{13}C NMR (126 MHz, $CDCl_3$) of compound **2b**.

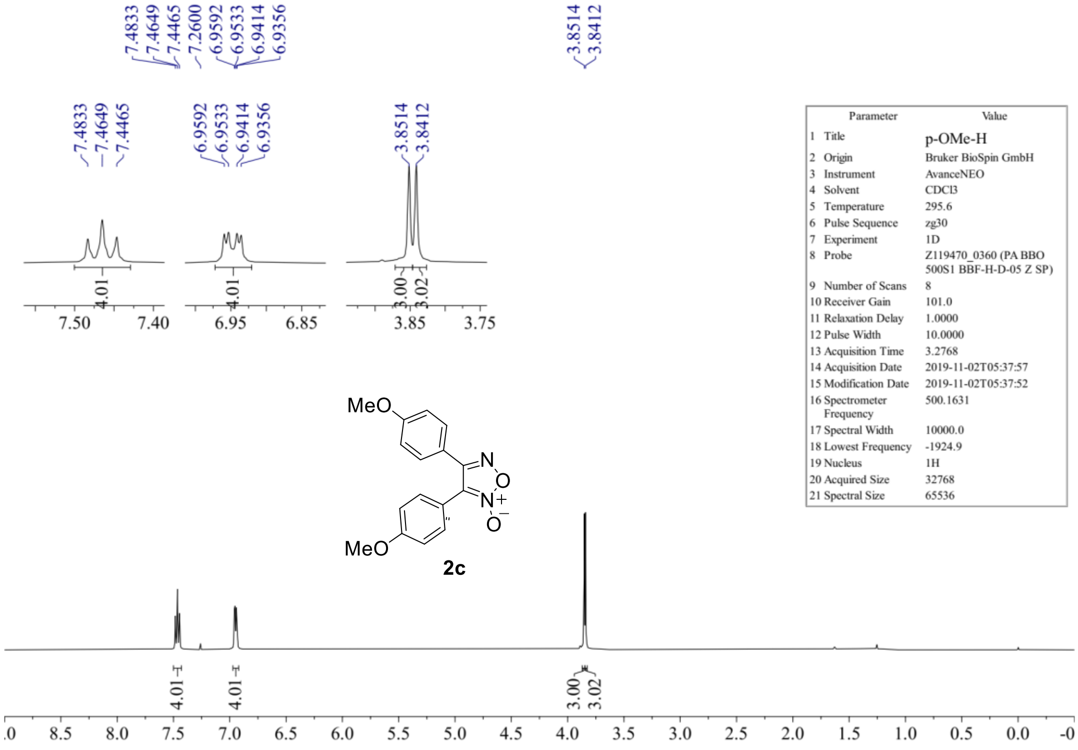

Figure S7. ^{1}H NMR (500 MHz, $CDCl_3$) of compound **2c**.

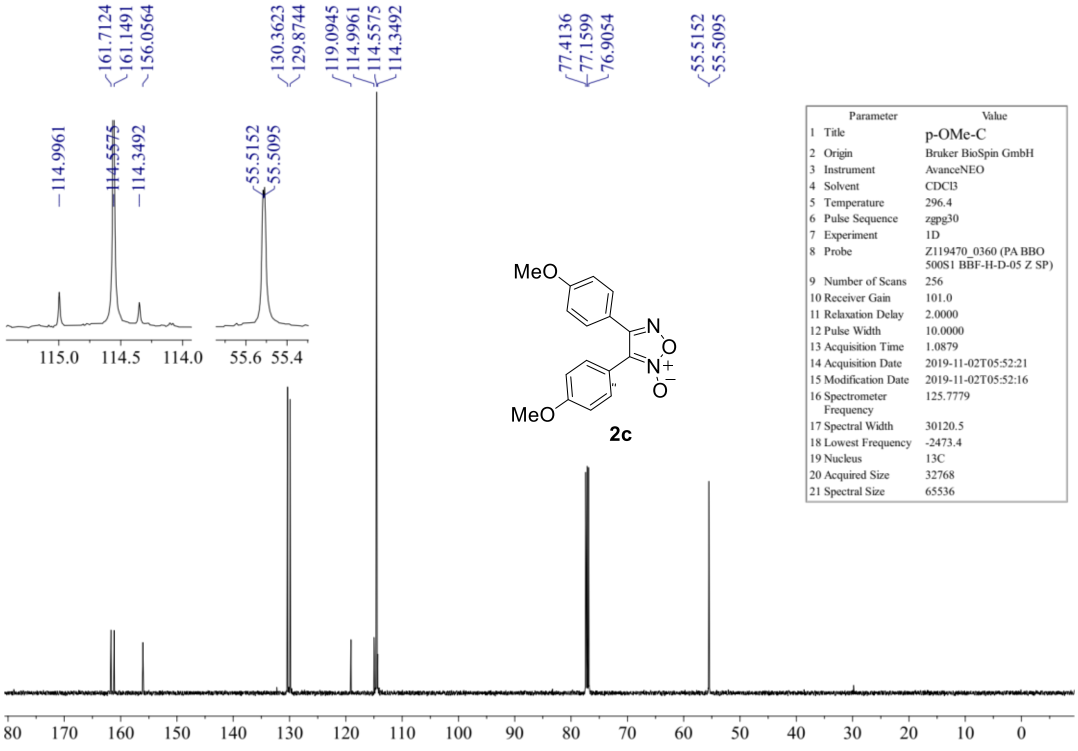

Figure S8. ^{13}C NMR (126 MHz, $CDCl_3$) of compound **2c**.

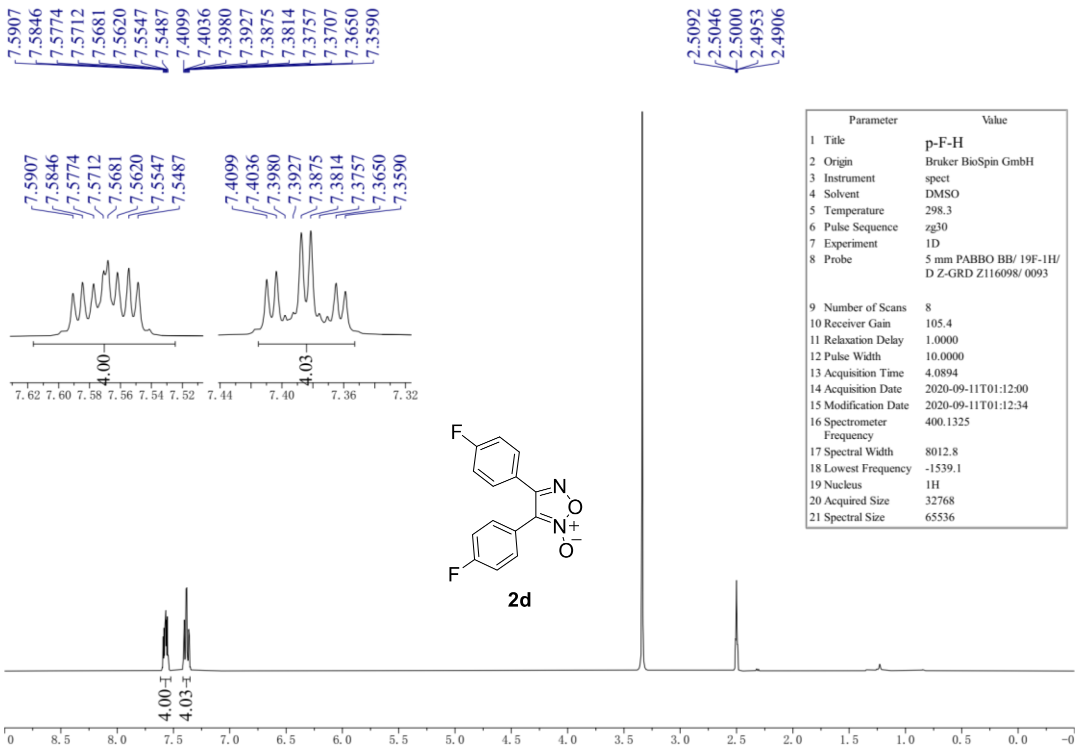

Figure S9. ^{1}H NMR (400 MHz, DMSO-d_6) of compound **2d**.

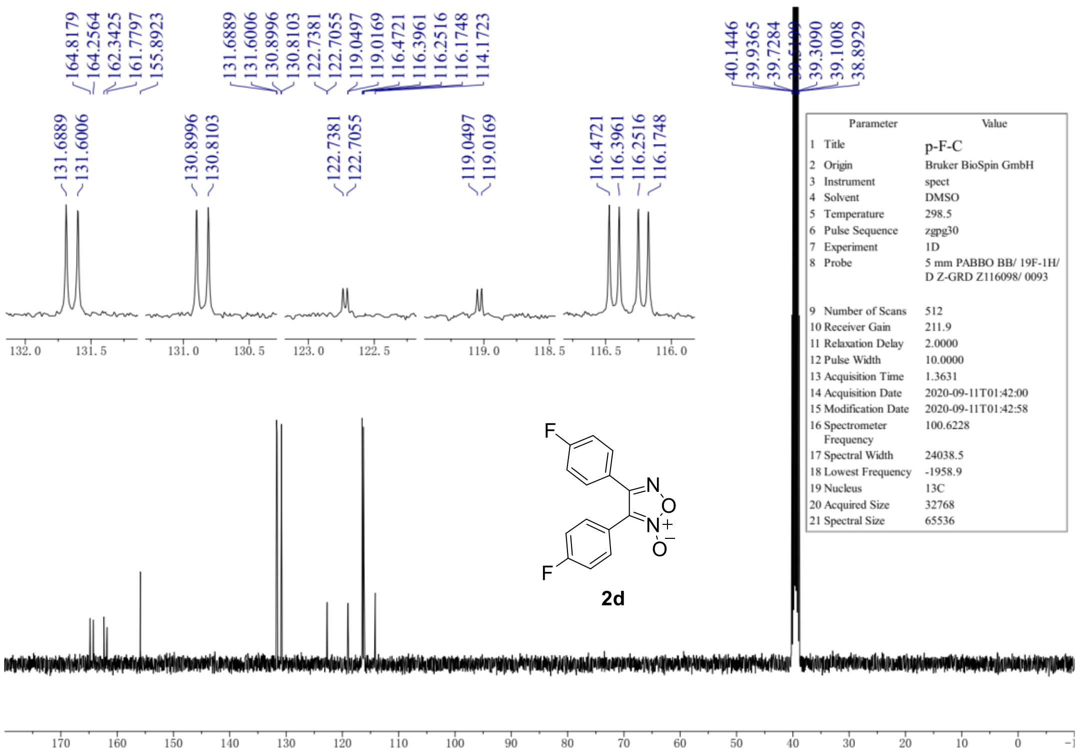

Figure S10. ^{13}C NMR (101 MHz, DMSO-d_6) of compound **2d**.

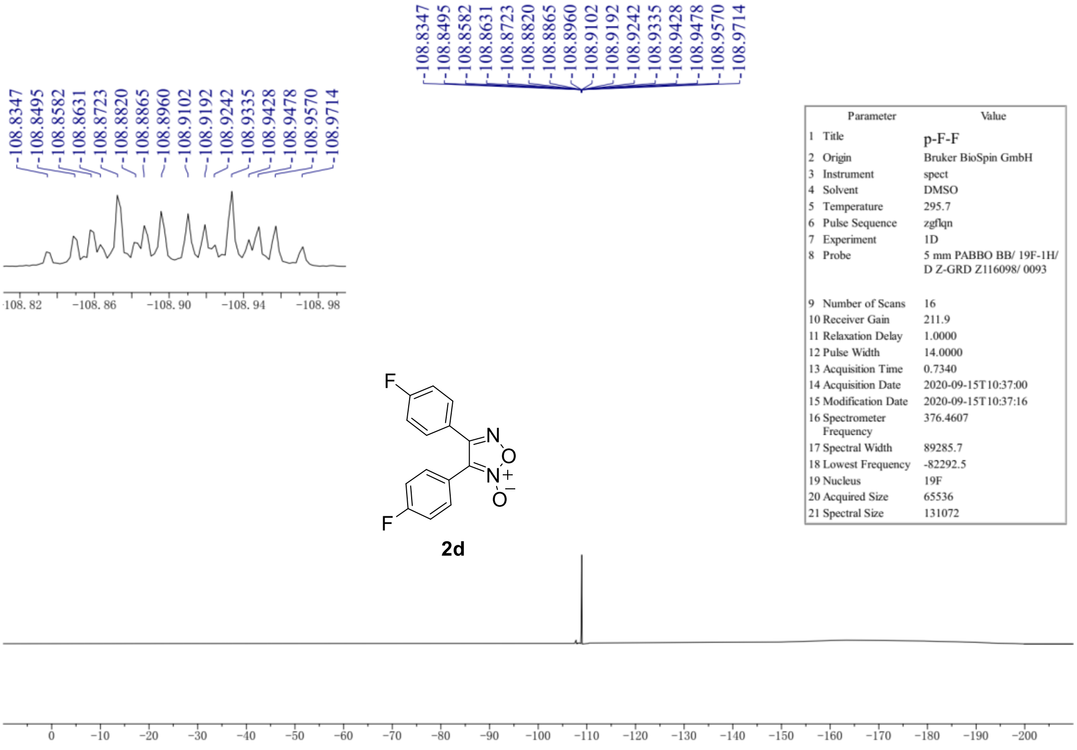

Figure S11. ^{19}F NMR (376 MHz, DMSO-d_6) of compound **2d**.

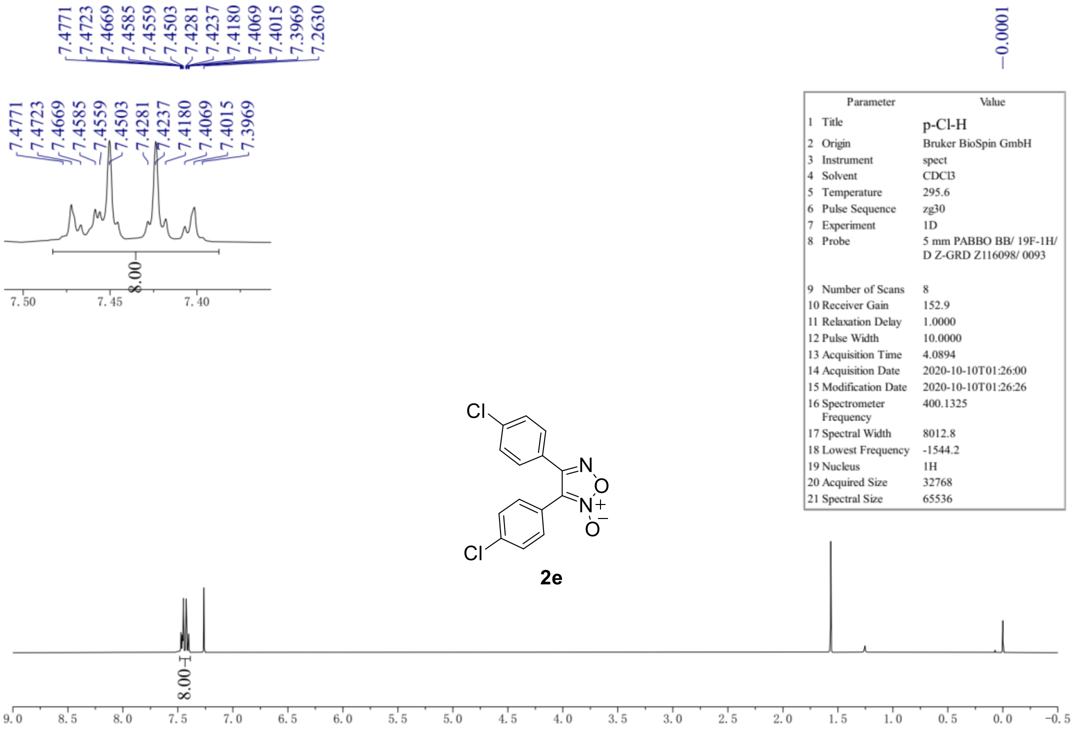

Figure S12. ^{1}H NMR (400 MHz, $CDCl_3$) of compound **2e**.

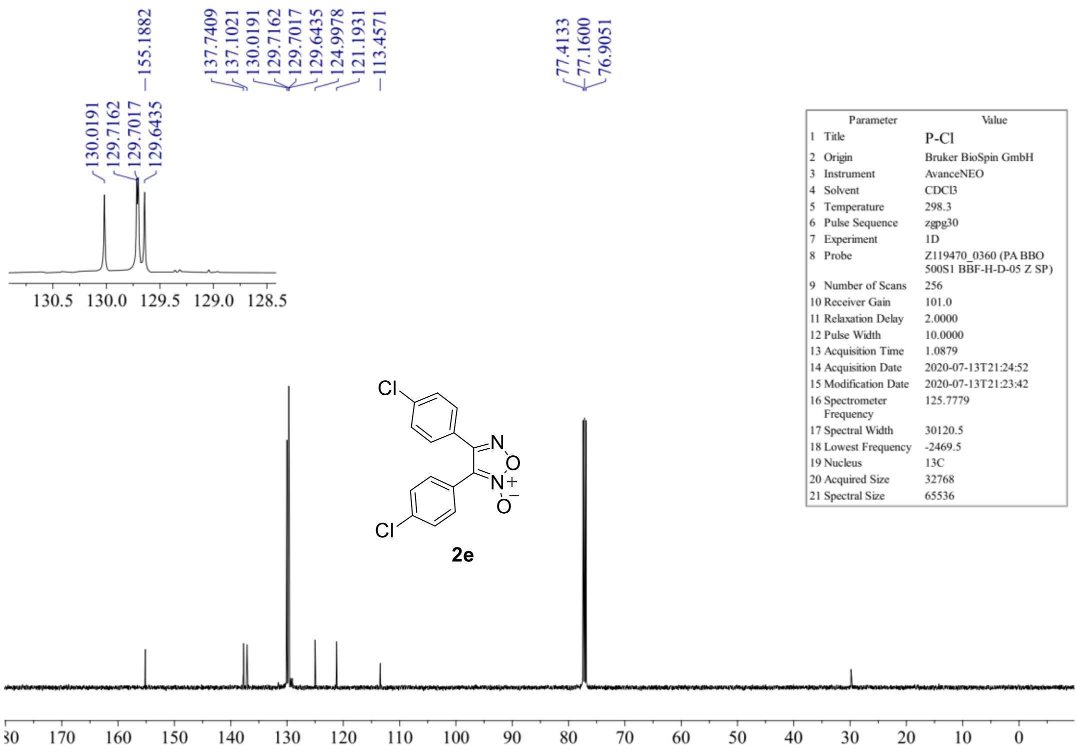

Figure S13. ^{13}C NMR (126 MHz, $CDCl_3$) of compound **2e**.

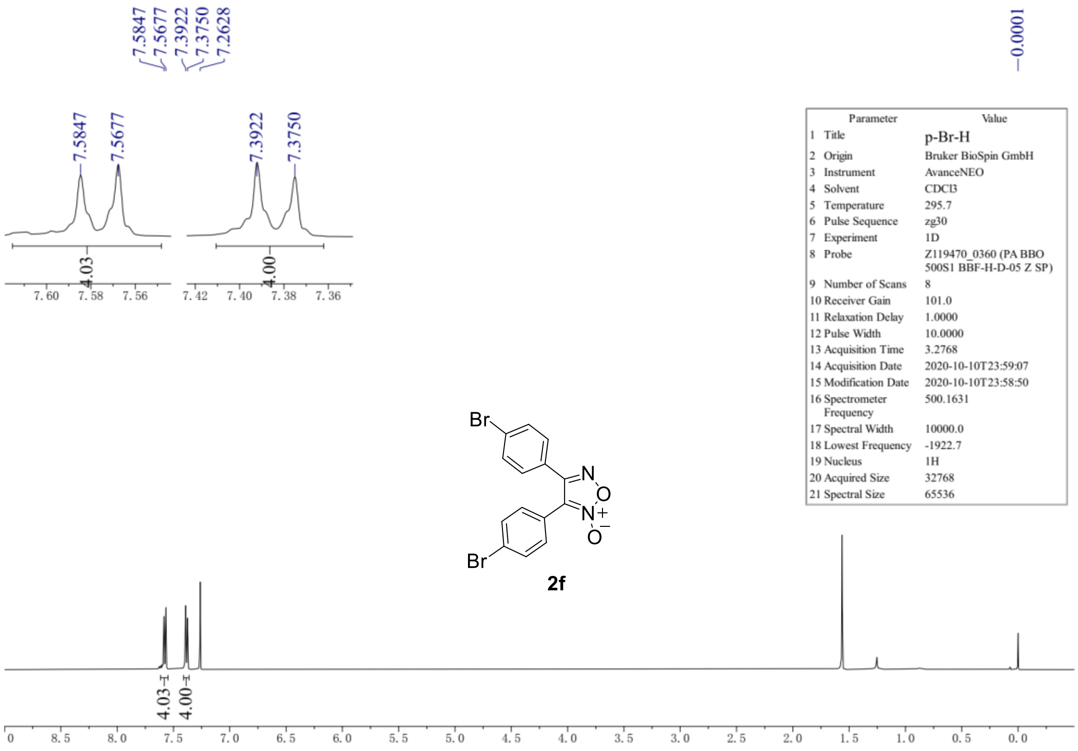

Figure S14. ^{1}H NMR (500 MHz, $CDCl_3$) of compound **2f**.

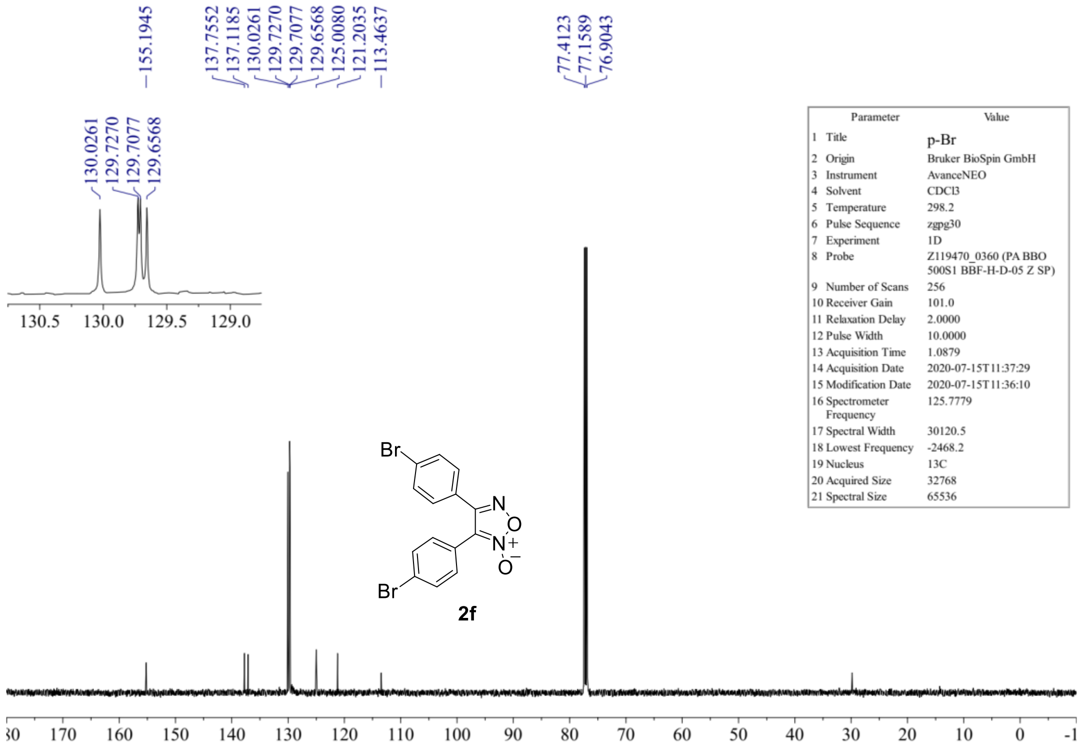

Figure S15. ^{13}C NMR (126 MHz, $CDCl_3$) of compound **2f**.

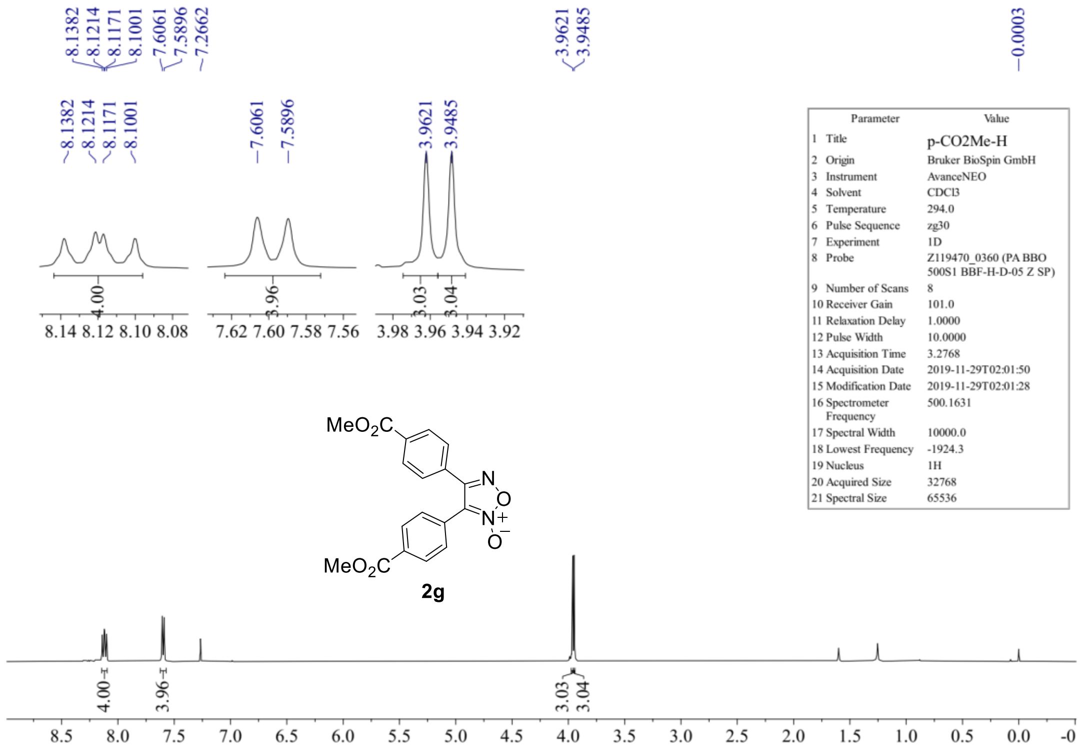

Figure S16. ^{1}H NMR (500 MHz, $CDCl_3$) of compound **2g**.

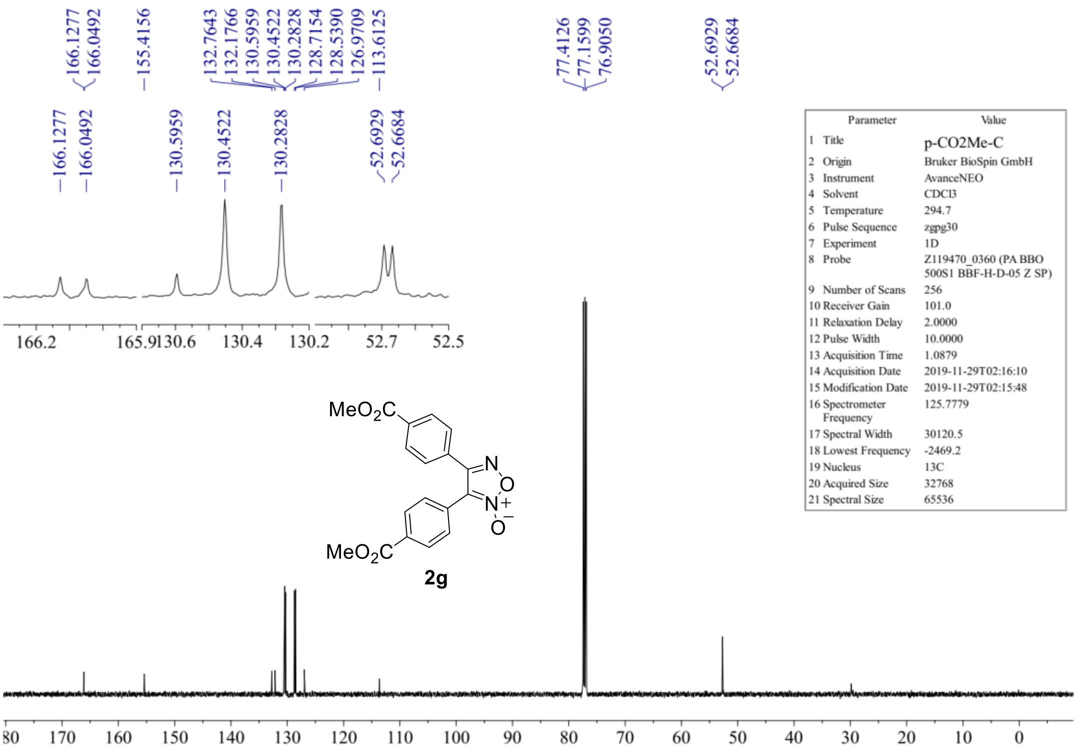

Figure S17. ^{13}C NMR (126 MHz, $CDCl_3$) of compound **2g**.

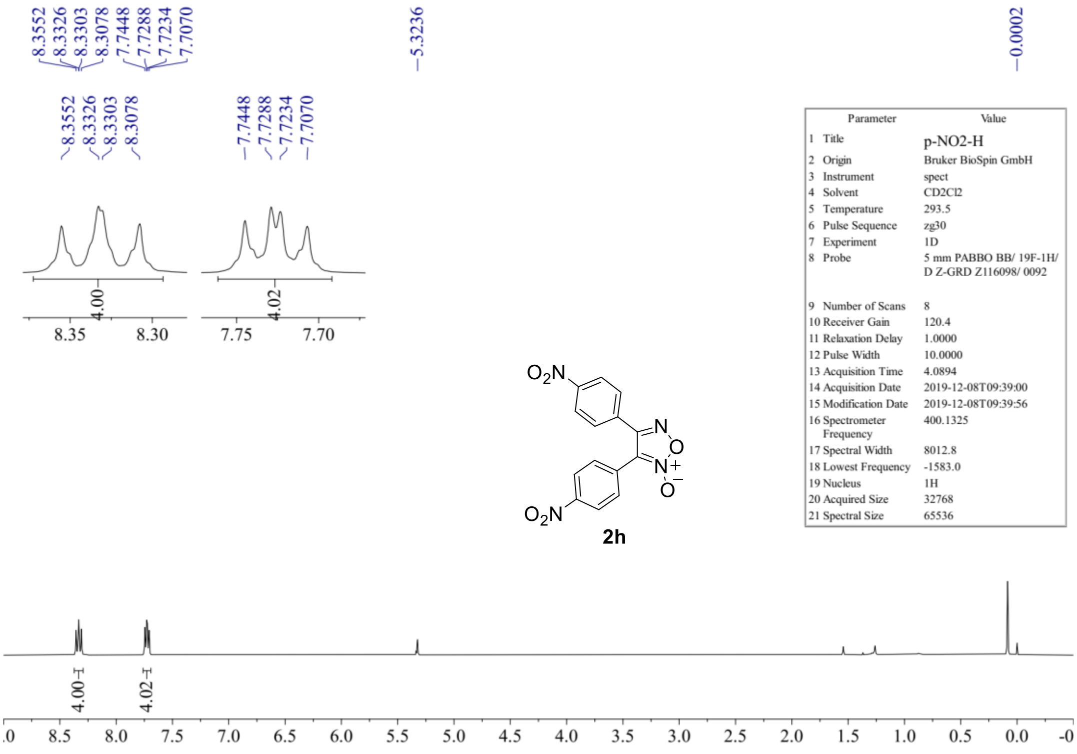

Figure S18. ^{1}H NMR (400 MHz, CD_2Cl_2) of compound **2h**.

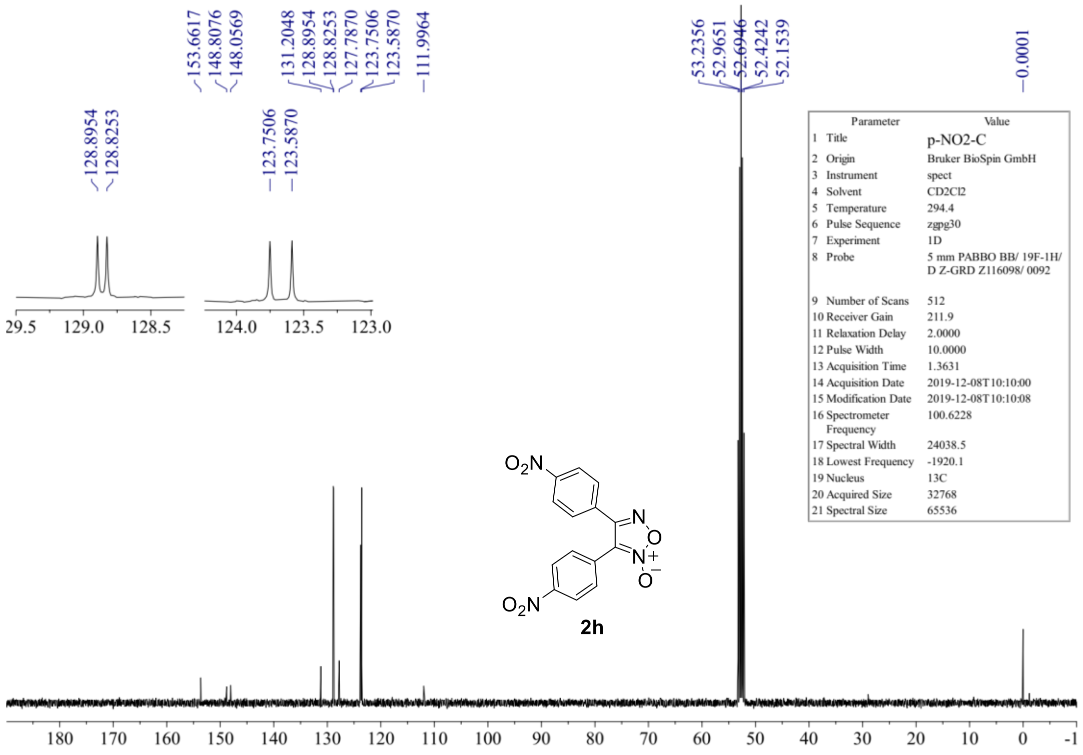

Figure S19. ^{13}C NMR (101 MHz, CD_2Cl_2) of compound **2h**.

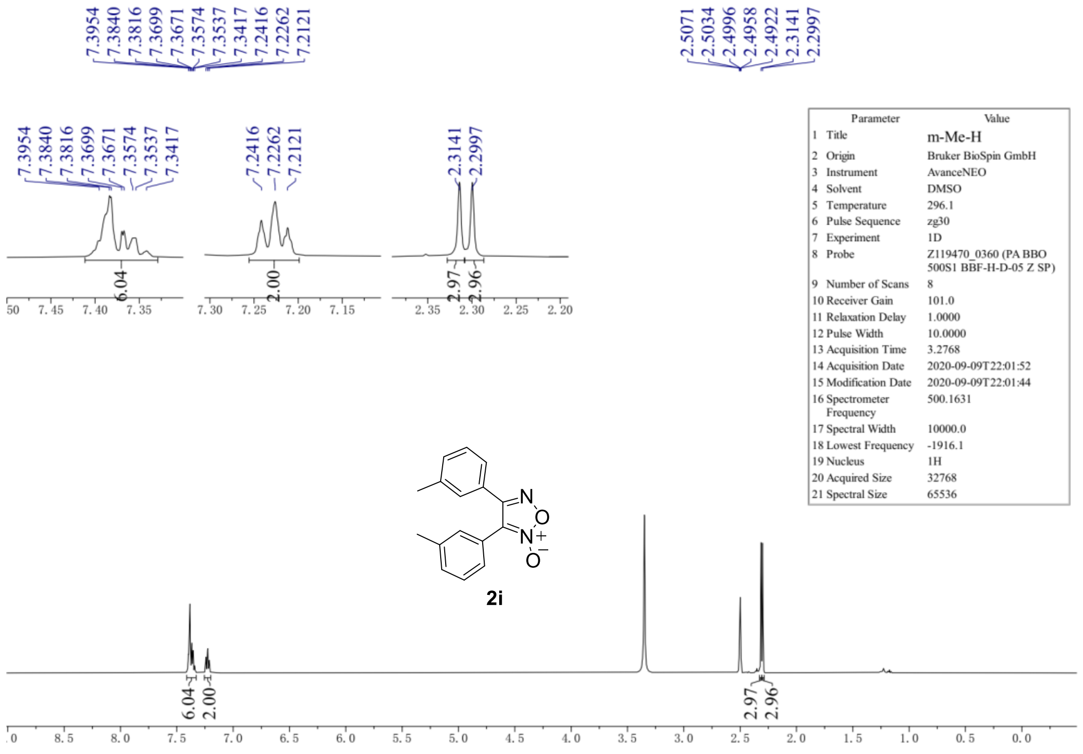

Figure S20. ^{1}H NMR (500 MHz, DMSO-d_6) of compound **2i**.

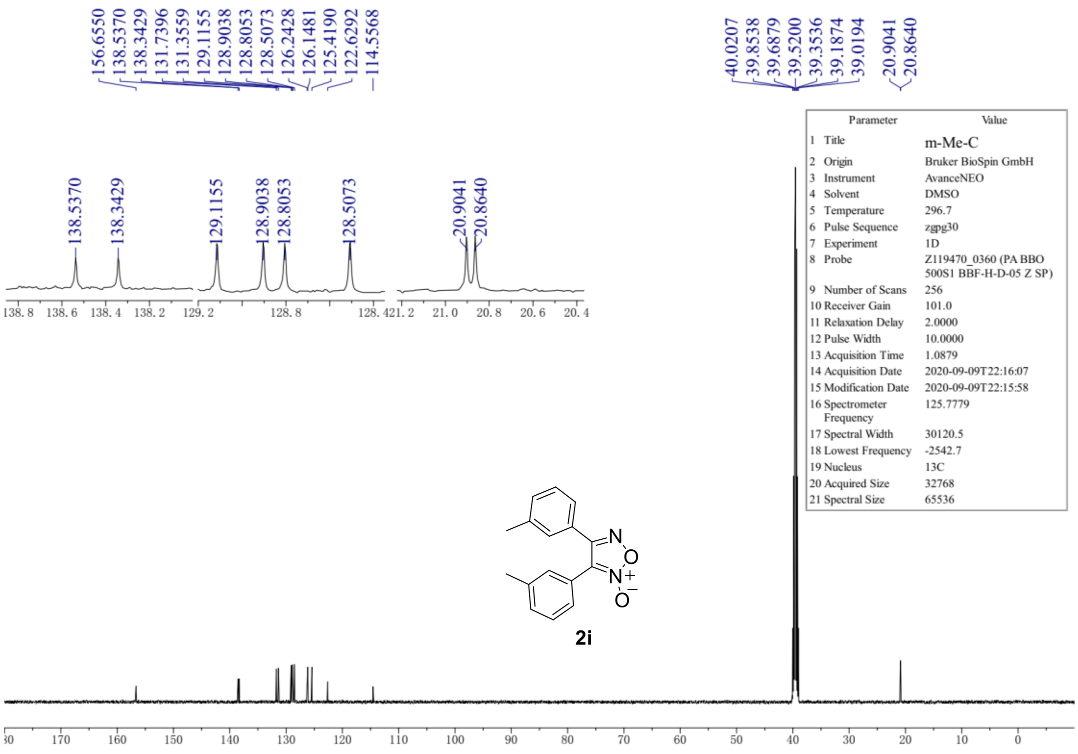

Figure S21. ^{13}C NMR (126 MHz, DMSO-d_6) of compound **2i**.

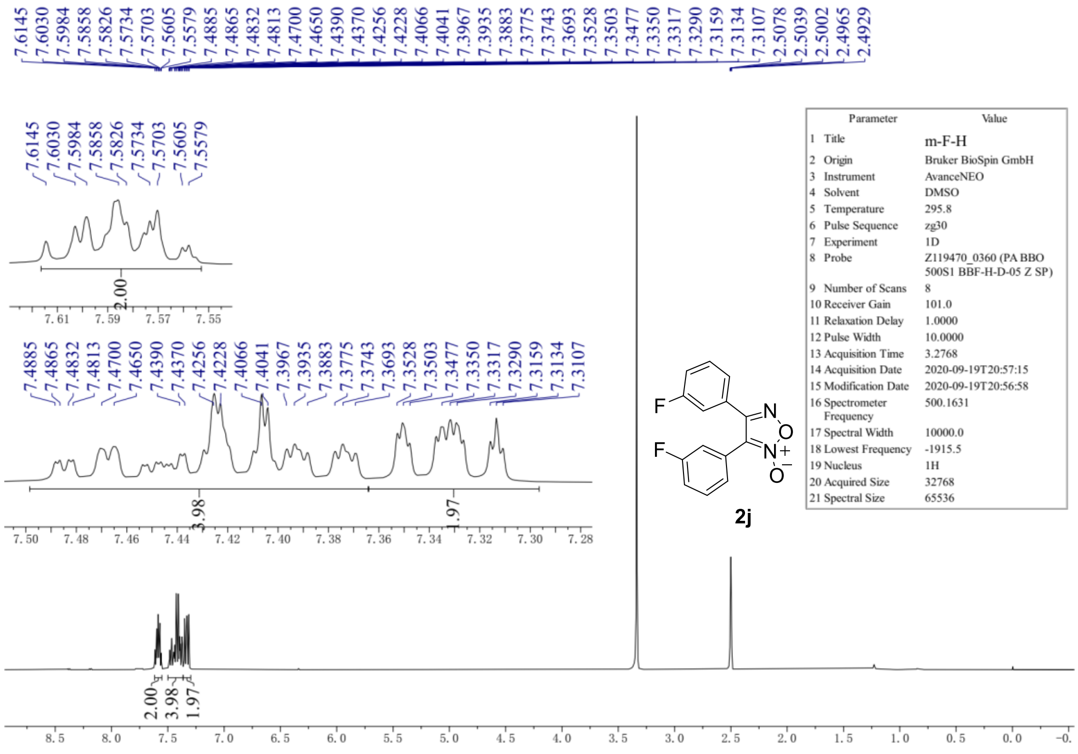

Figure S22. ^{1}H NMR (500 MHz, DMSO-d_6) of compound **2j**.

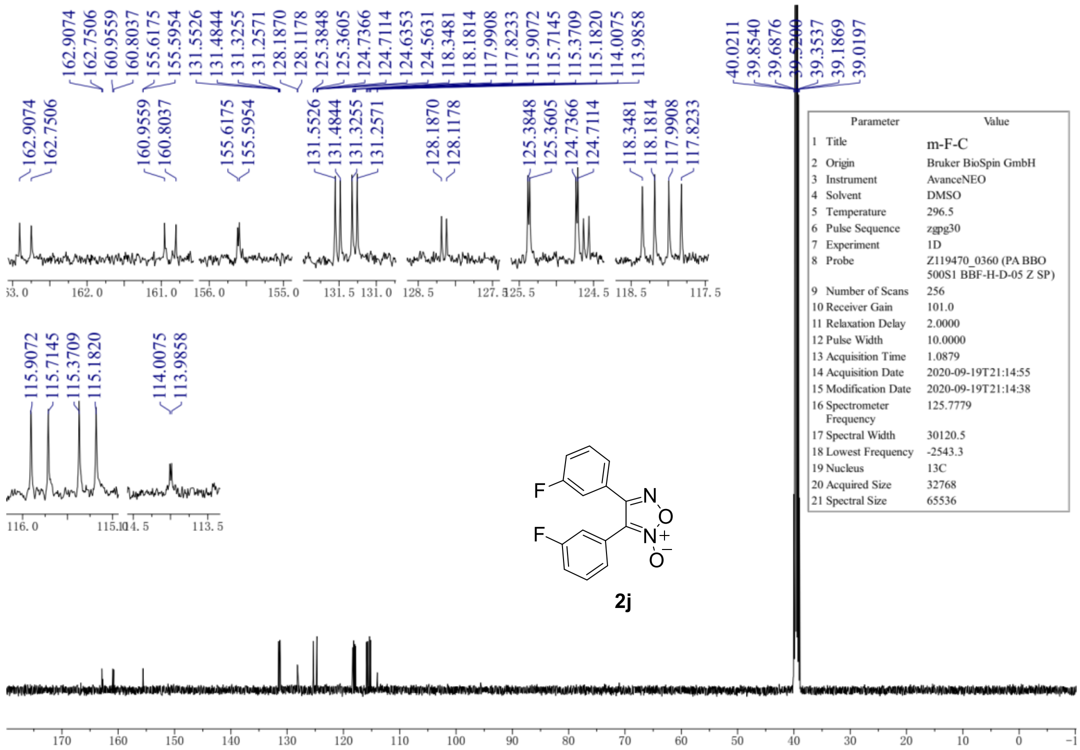

Figure S23. ^{13}C NMR (126 MHz, DMSO-d_6) of compound **2j**.

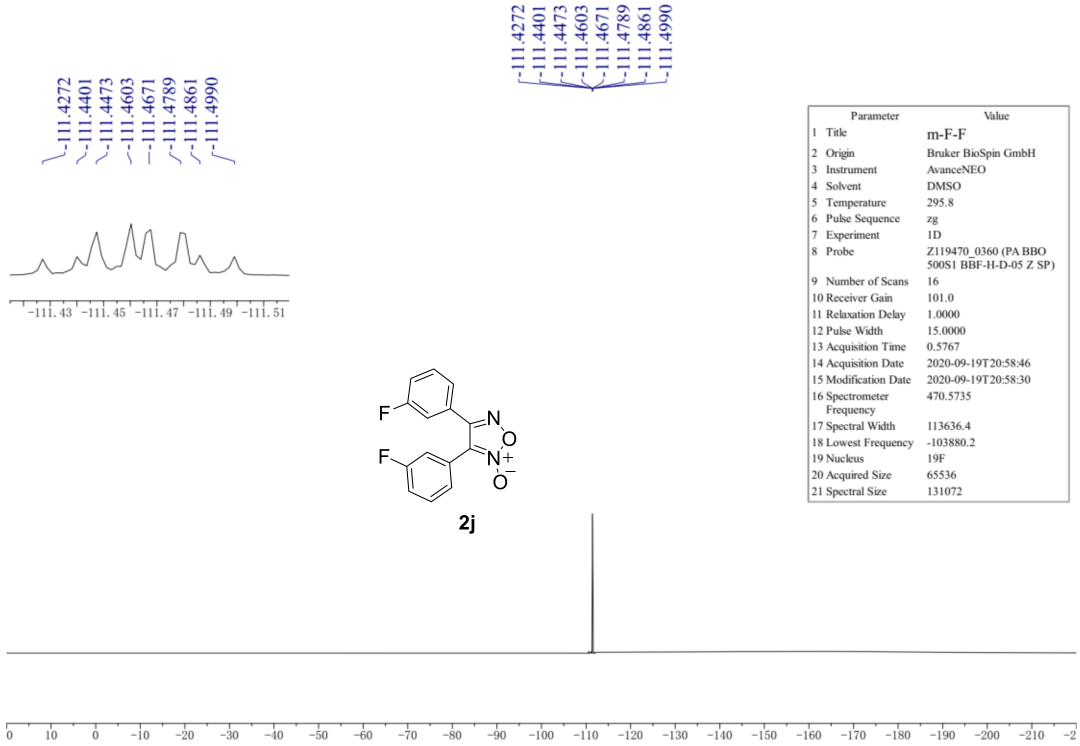

Figure S24. ^{19}F NMR (471 MHz, DMSO-d_6) of compound **2j**.

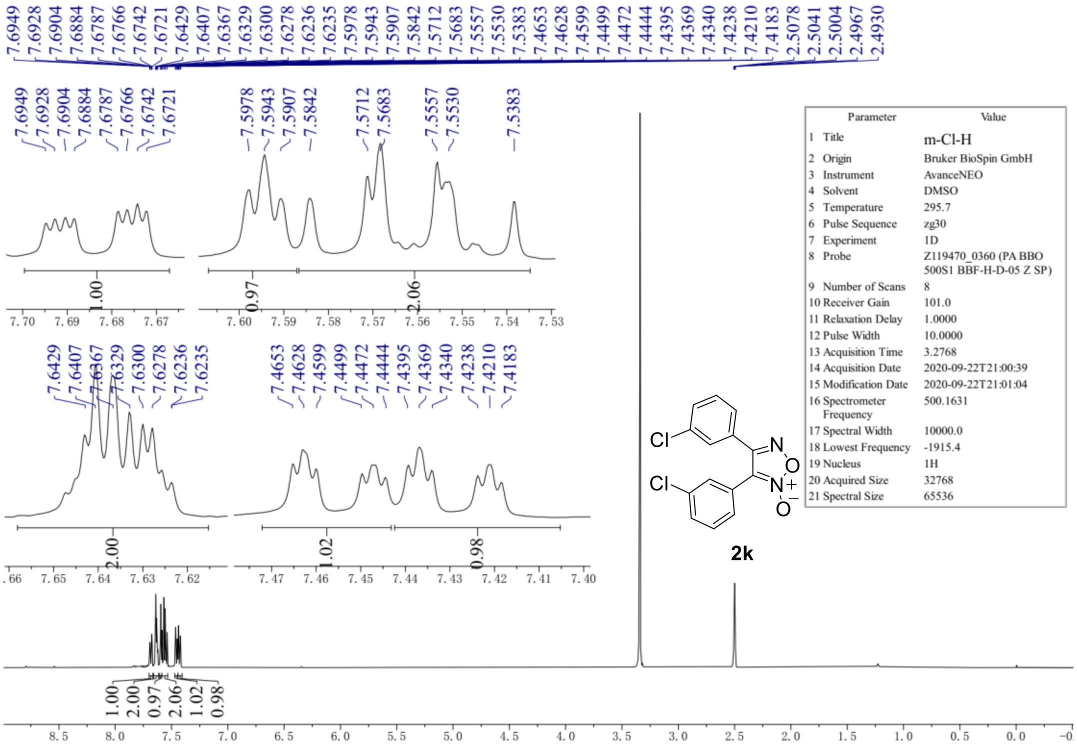

Figure S25. ^{1}H NMR (500 MHz, DMSO-d_6) of compound **2k**.

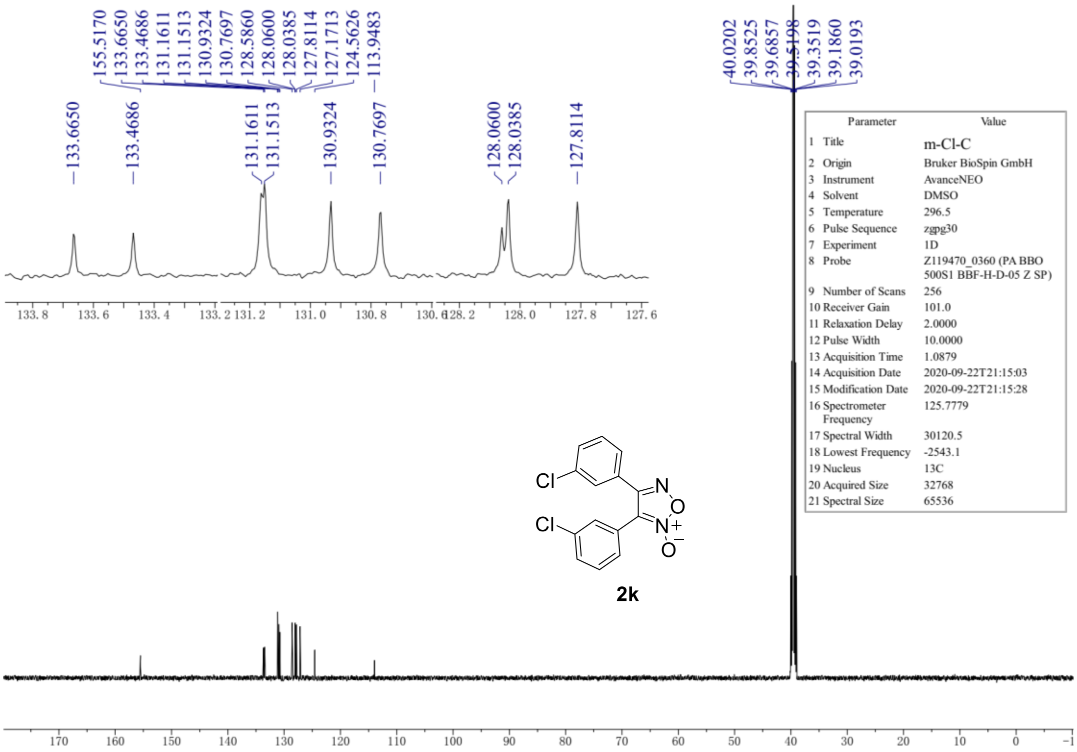

Figure S26. ^{13}C NMR (126 MHz, DMSO-d_6) of compound **2k**.

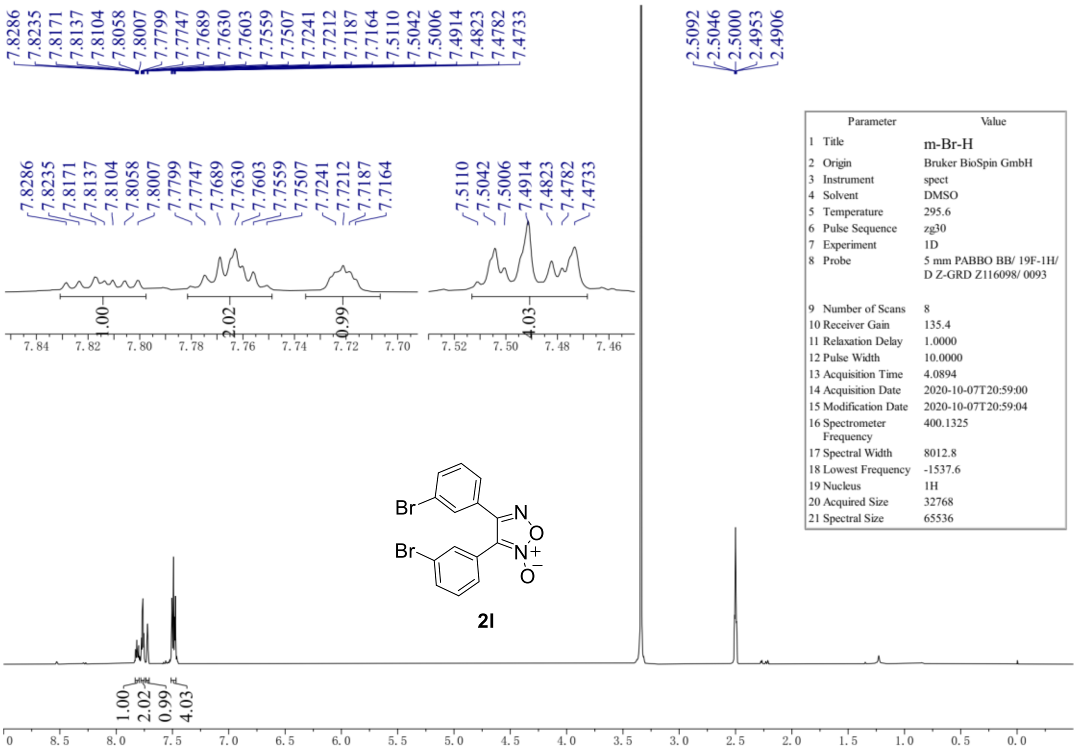

Figure S27. ^{1}H NMR (400 MHz, DMSO-d_6) of compound **2l**.

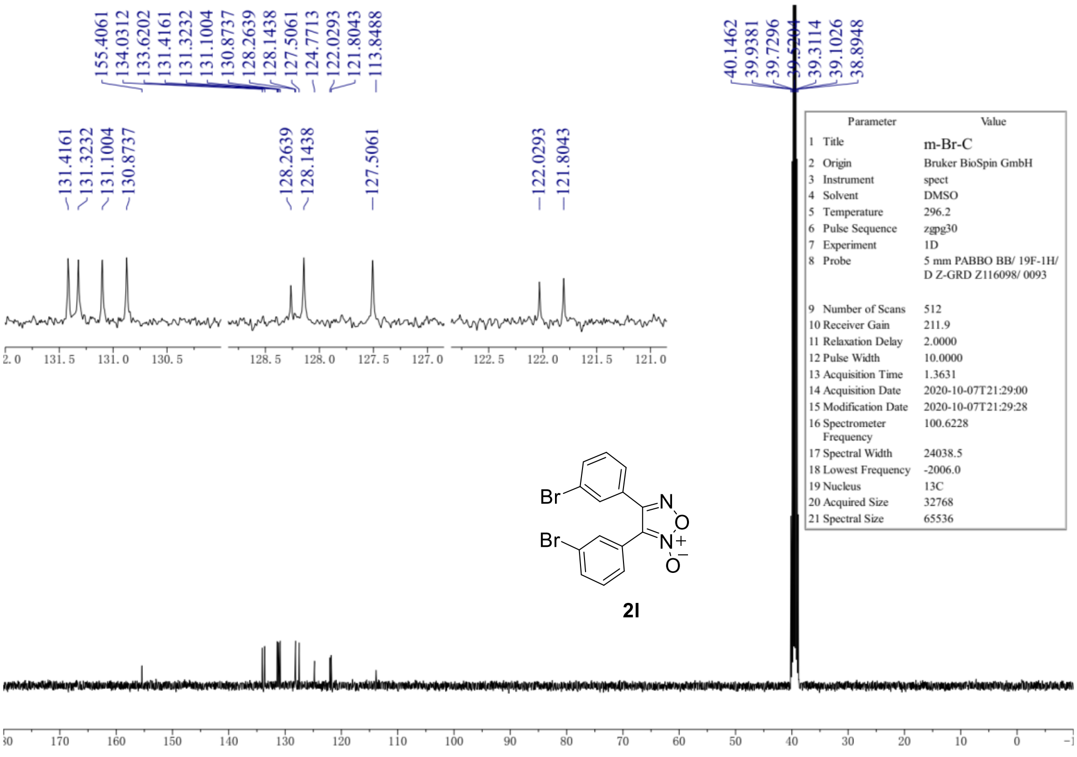

Figure S28. ^{13}C NMR (101 MHz, DMSO-d_6) of compound **2l**.

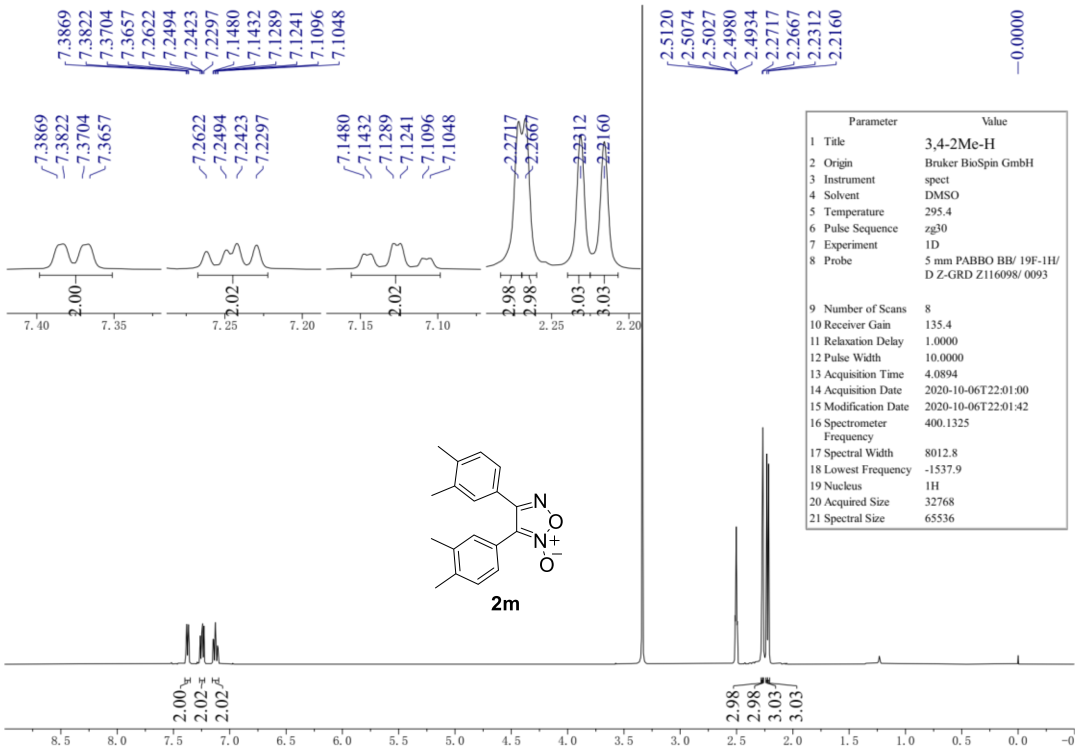

Figure S29. ^{1}H NMR (400 MHz, DMSO-d_6) of compound **2m**.

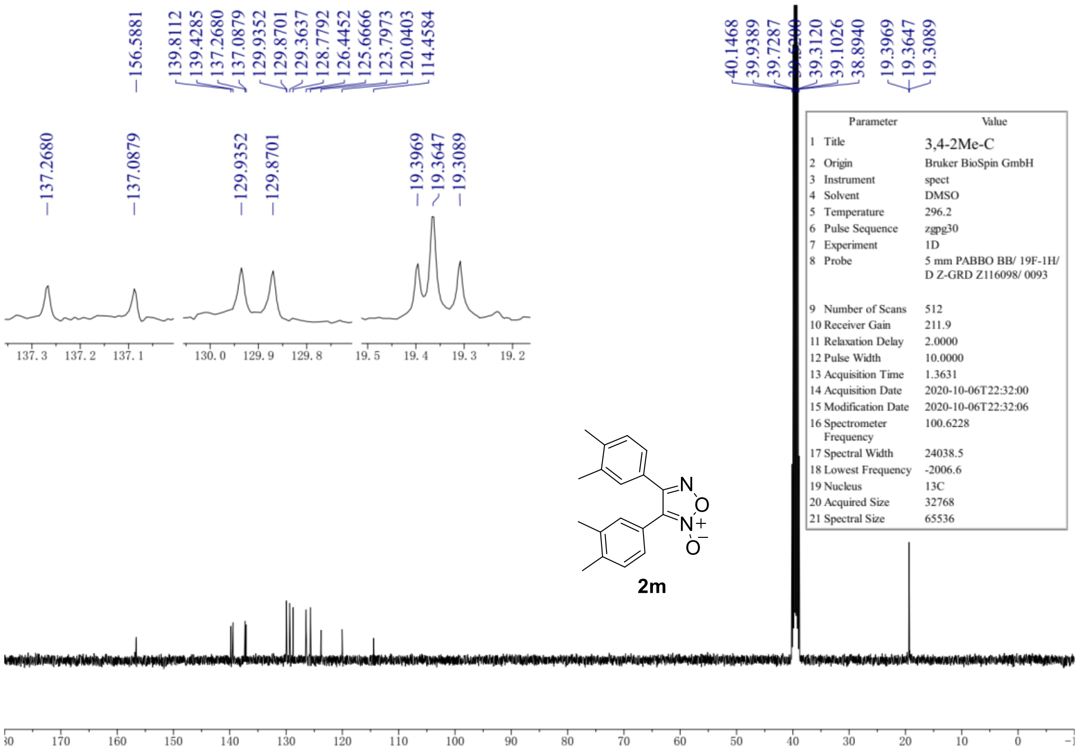

Figure S30. ^{13}C NMR (101 MHz, DMSO-d_6) of compound **2m**.

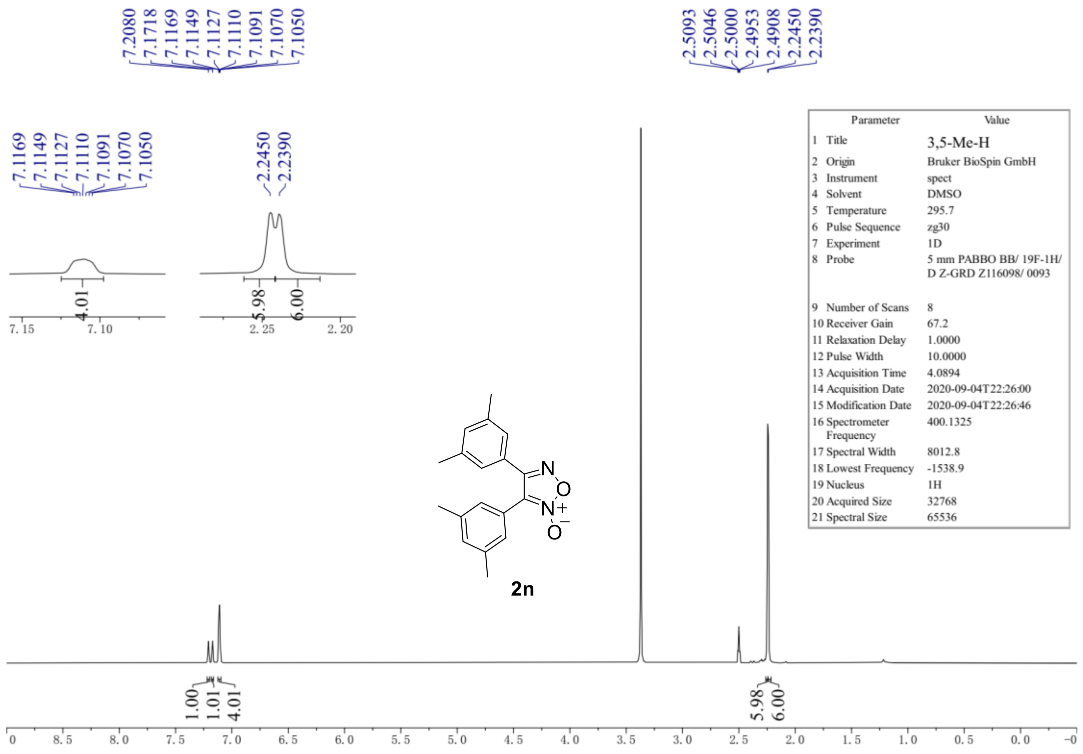

Figure S31. ^{1}H NMR (400 MHz, DMSO-d_6) of compound **2n**.

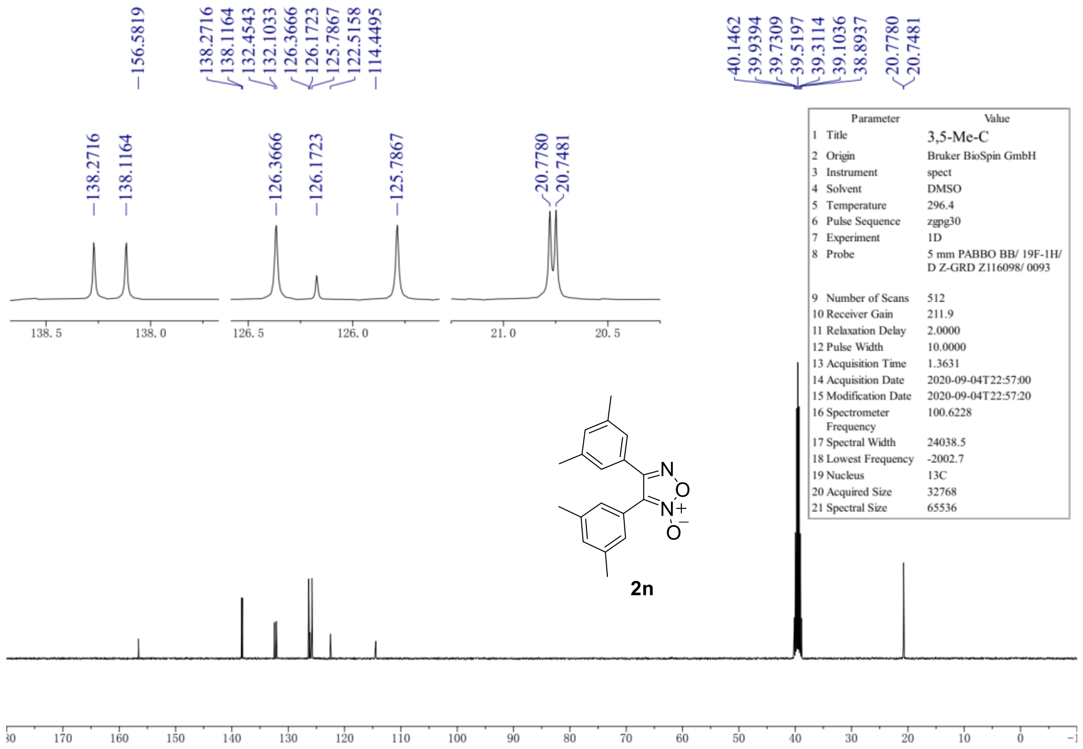

Figure S32. ^{13}C NMR (101 MHz, DMSO-d_6) of compound **2n**.

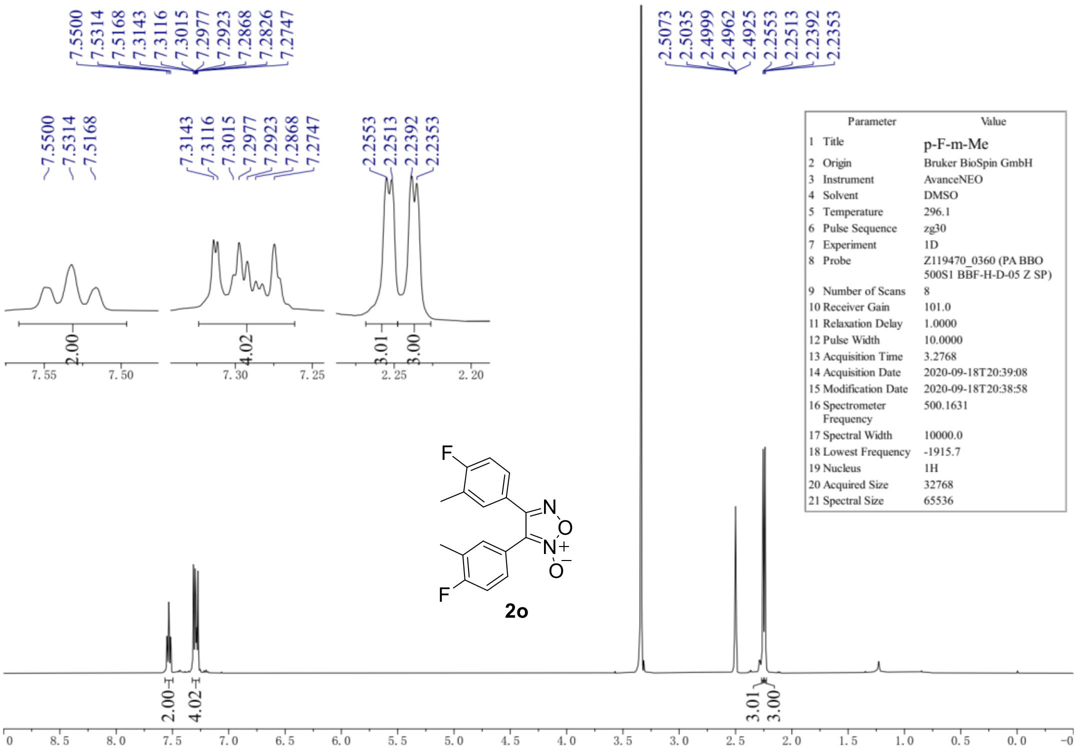

Figure S33. ^{1}H NMR (500 MHz, DMSO-d_6) of compound **2o**.

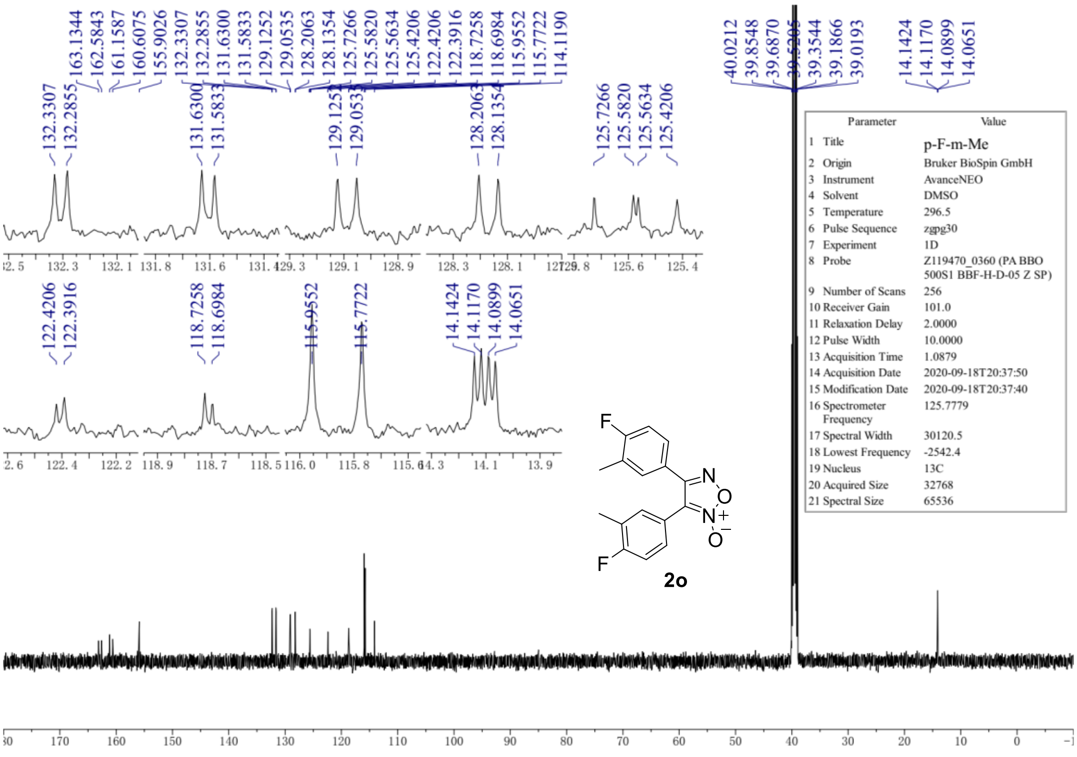

Figure S34. ^{13}C NMR (126 MHz, DMSO-d_6) of compound **2o**.

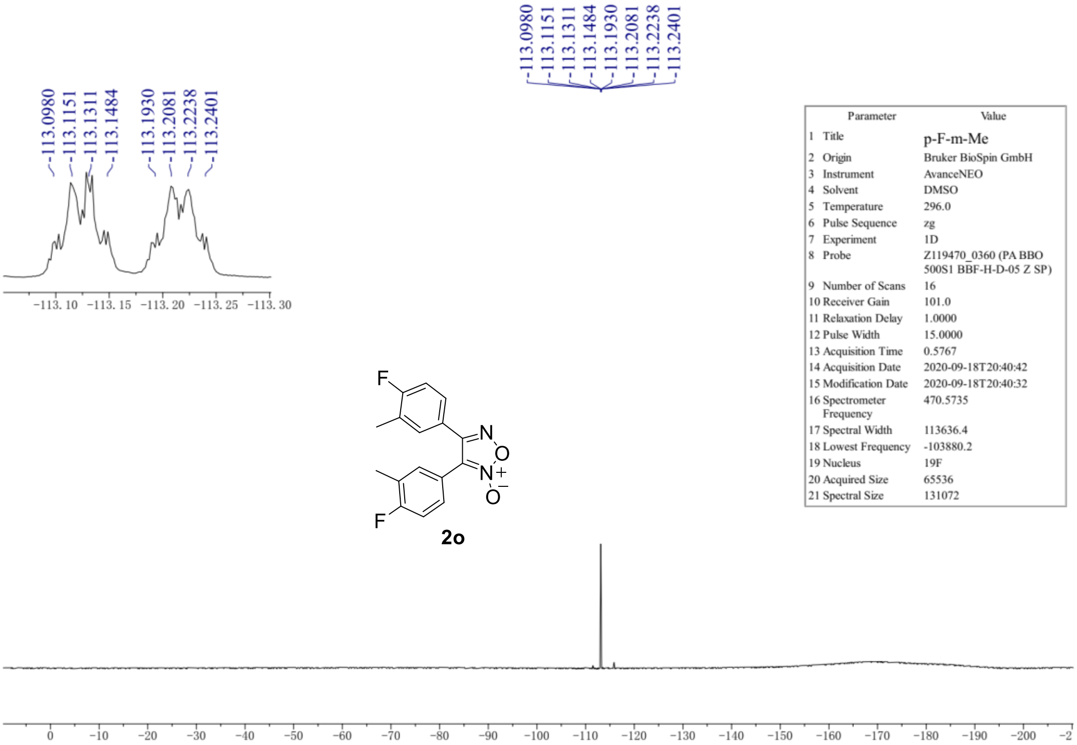

Figure S35. ^{19}F NMR (471 MHz, DMSO-d_6) spectrum of compound **2o**.

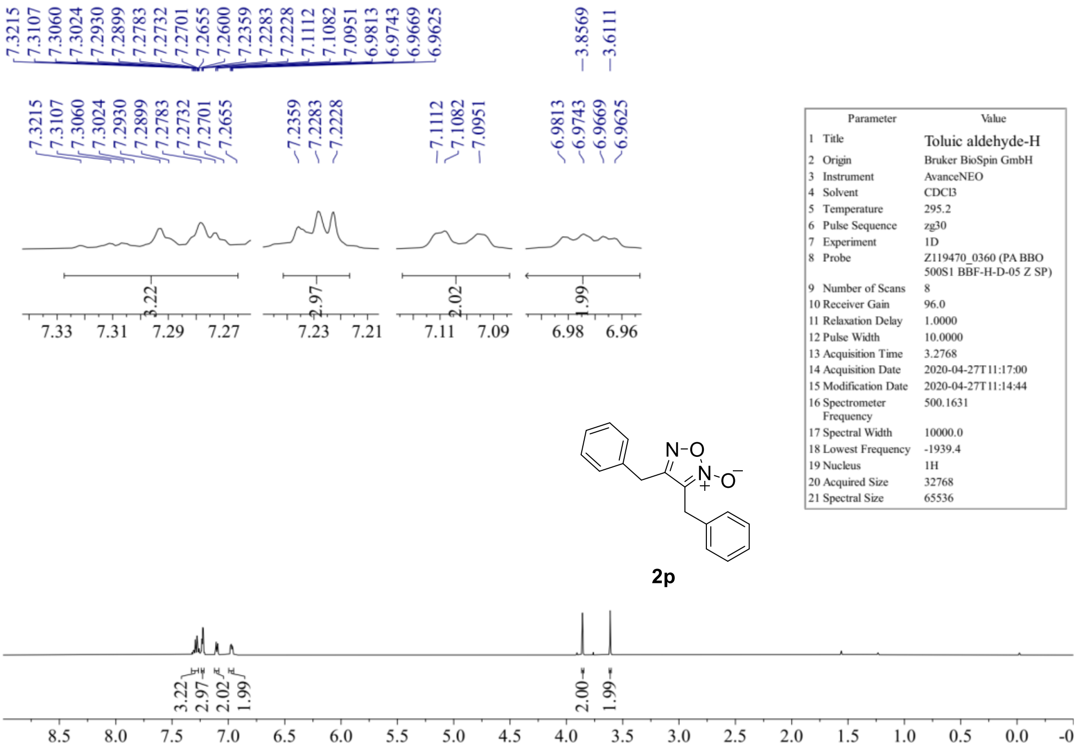

Figure S36. ^{1}H NMR (500 MHz, $CDCl_3$) of compound **2p**.

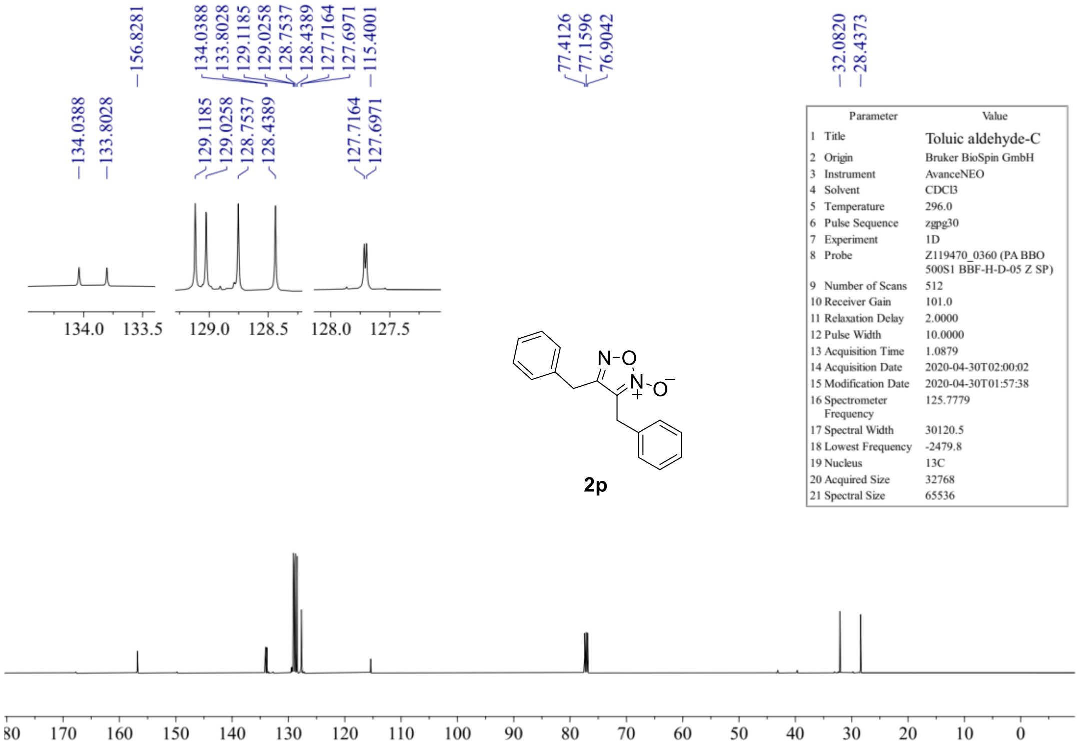

Figure S37. ^{13}C NMR (126 MHz, $CDCl_3$) of compound **2p**.

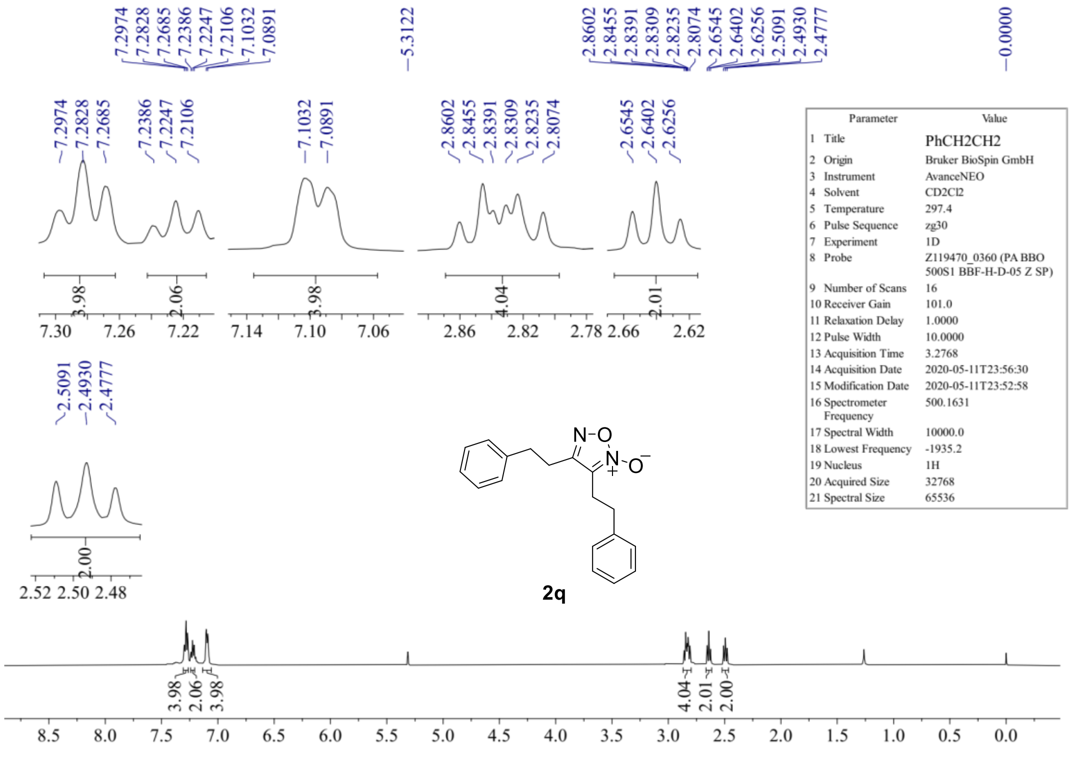

Figure S38. ^{1}H NMR (500 MHz, CD_2Cl_2) of compound **2q**.

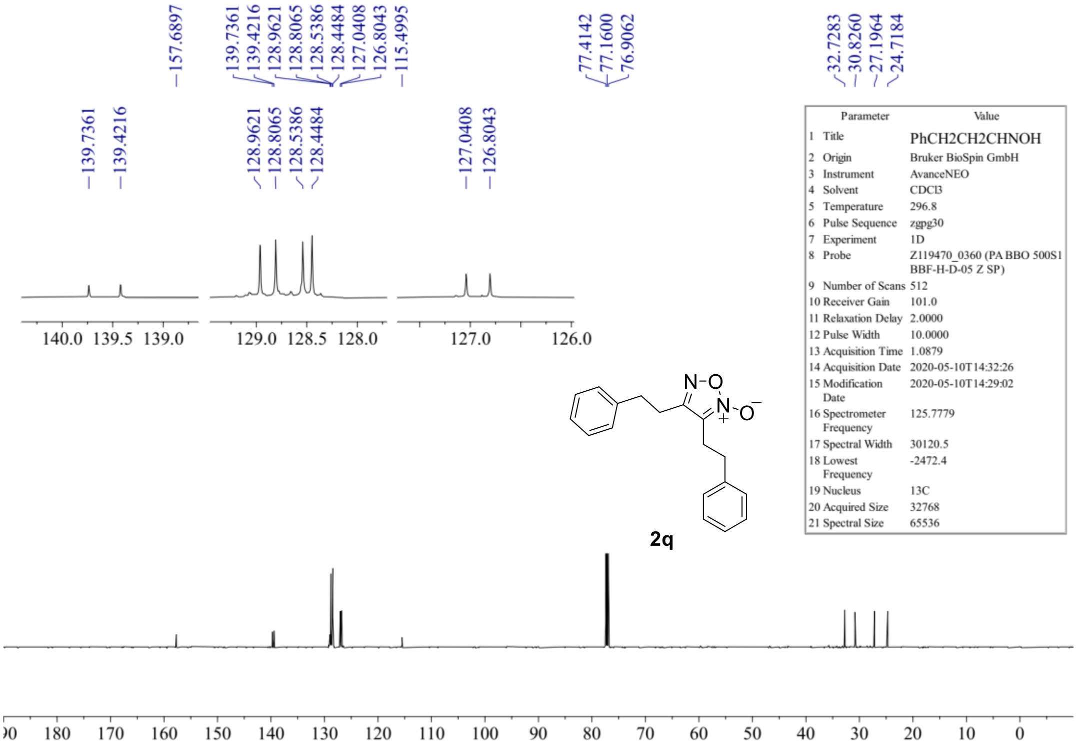

Figure S39. ^{13}C NMR (126 MHz, CD_2Cl_2) of compound **2q**.

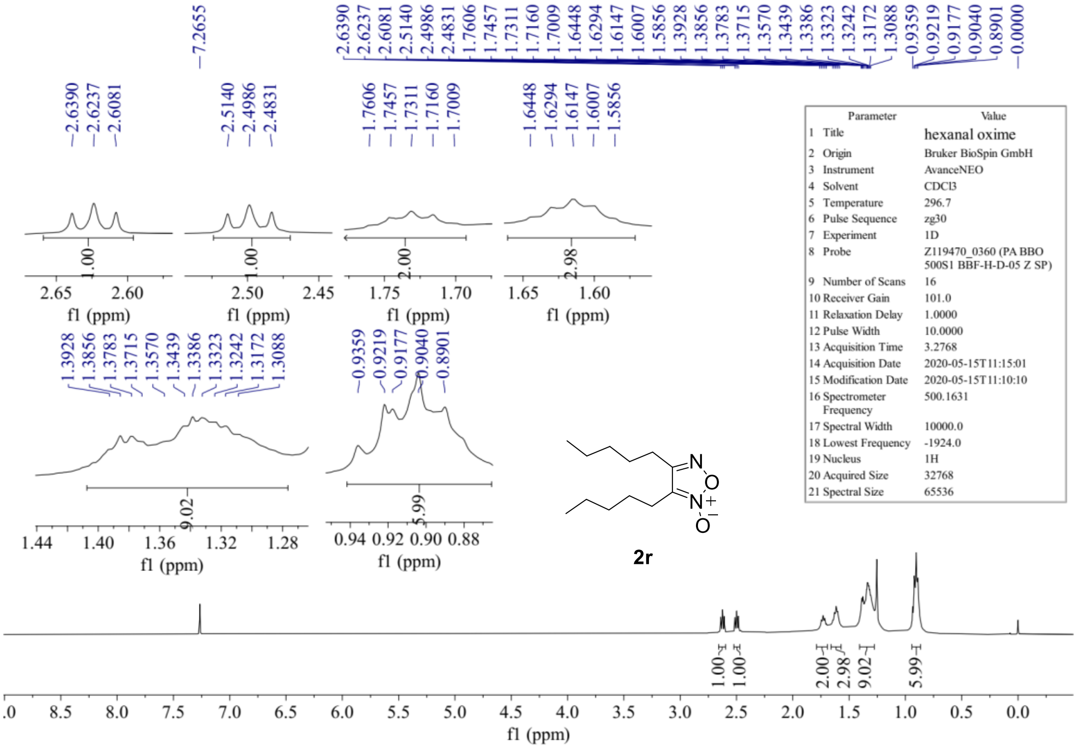

Figure S40. ^{1}H NMR (500 MHz, $CDCl_3$) of compound **2r**.

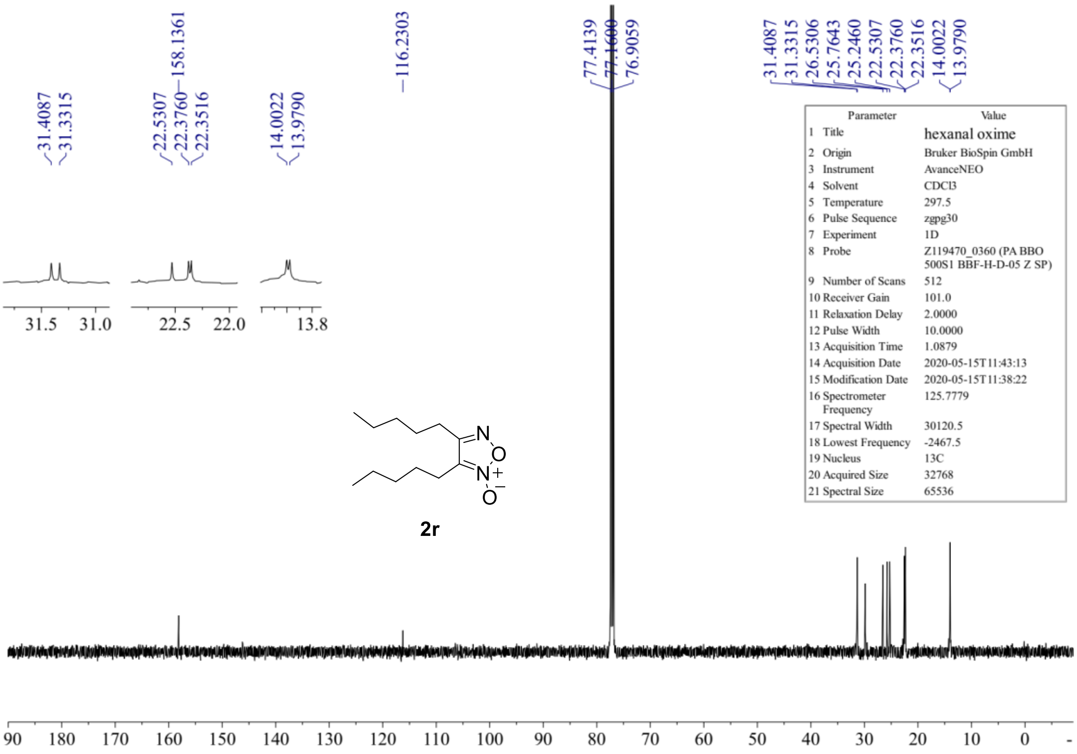

Figure S41. ^{13}C NMR (126 MHz, $CDCl_3$) of compound **2r**.

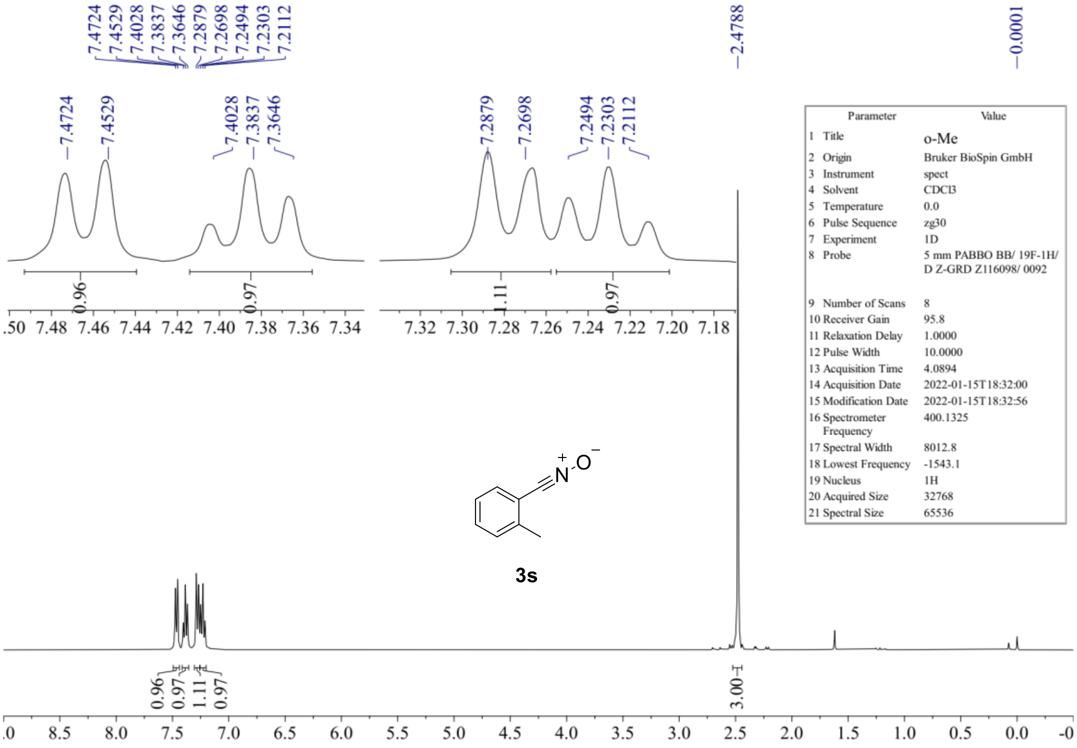

Figure S42. ^{1}H NMR (400 MHz, $CDCl_3$) of compound **3s**.

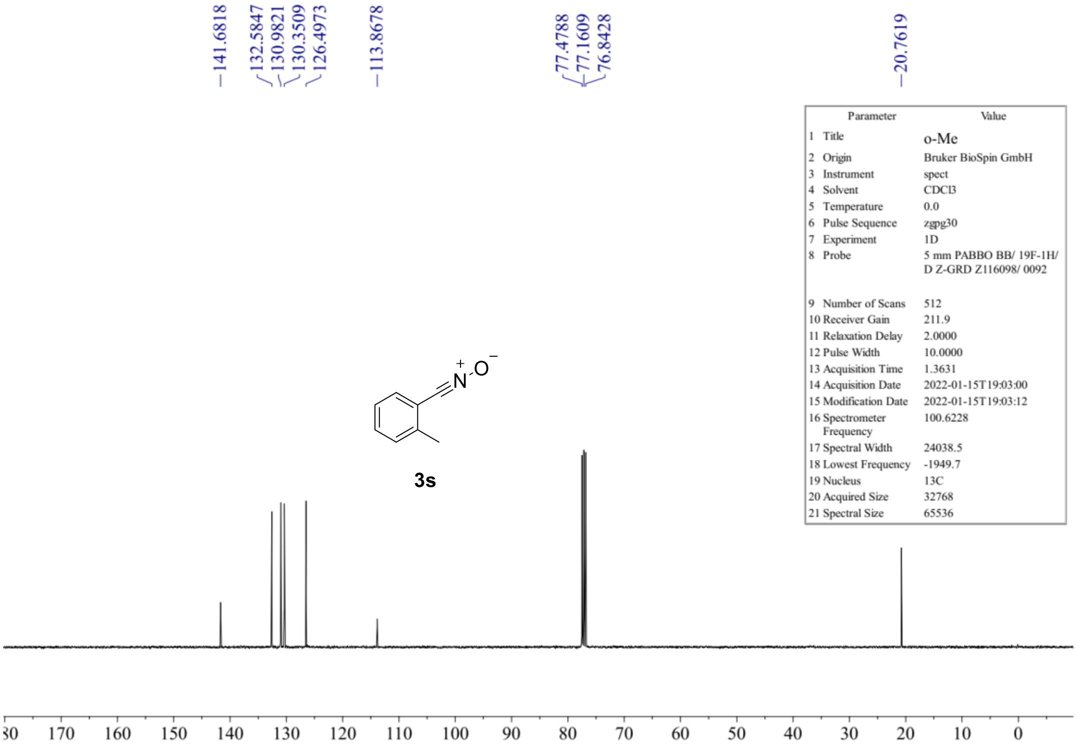

Figure S43. ^{13}C NMR (101 MHz, $CDCl_3$) of compound **3s**.

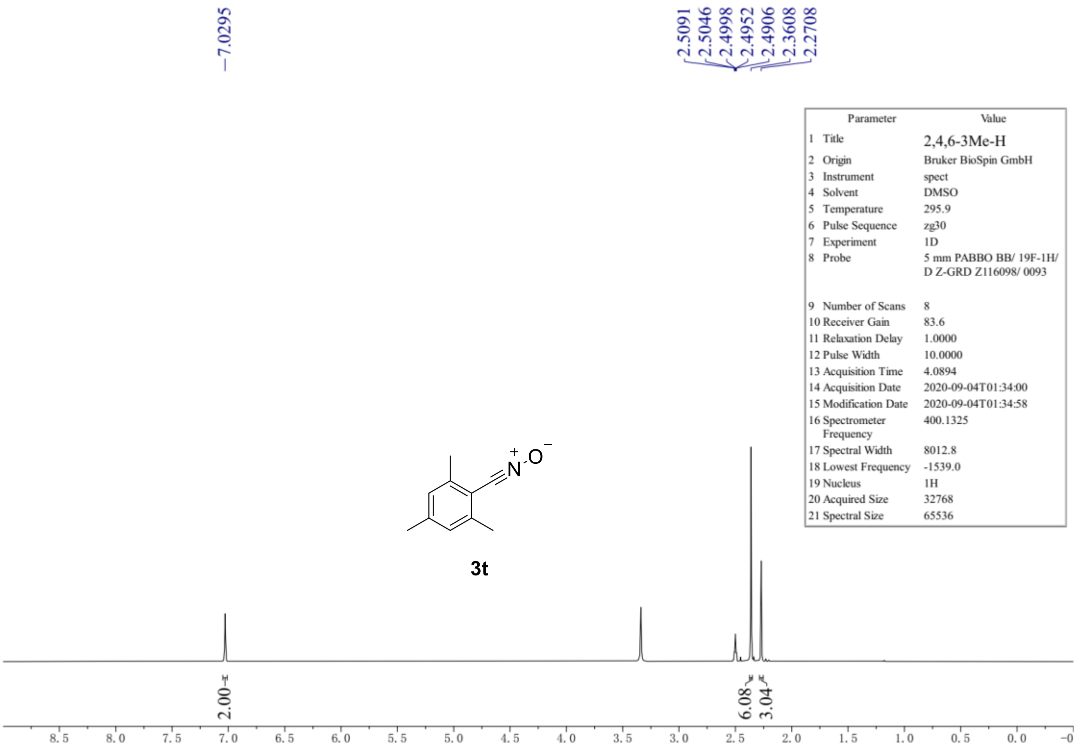

Figure S44. ^{1}H NMR (400 MHz, DMSO-d_6) of compound **3t**.

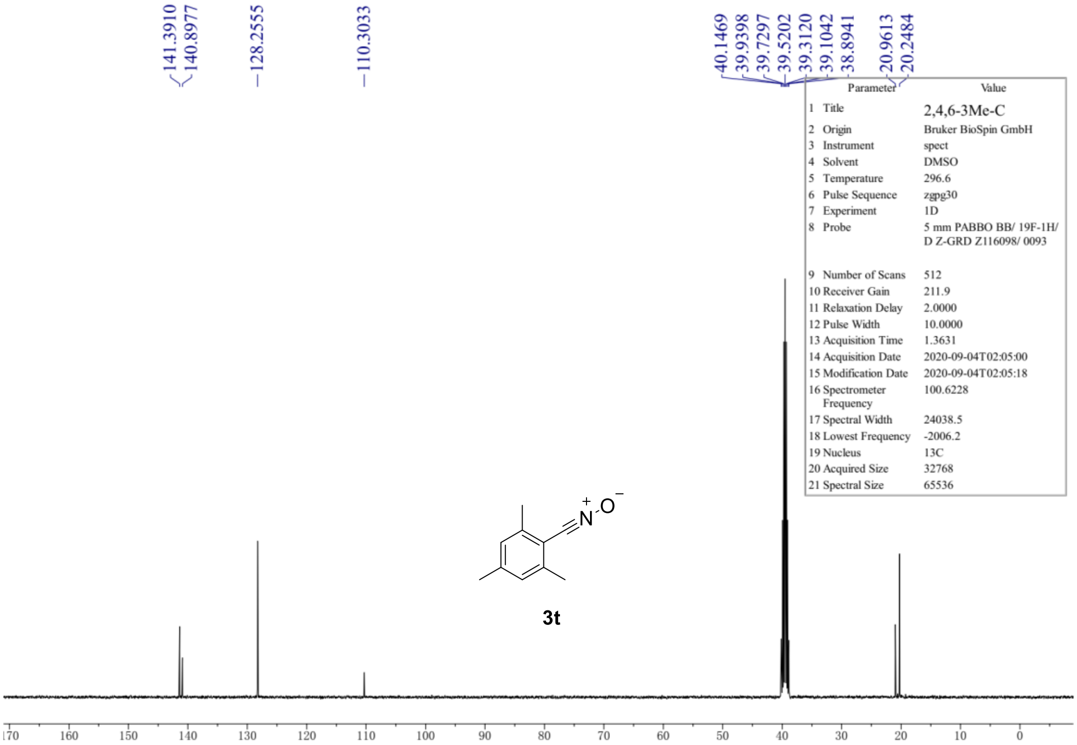

Figure S45. ^{13}C NMR (101 MHz, DMSO-d_6) of compound **3t**.

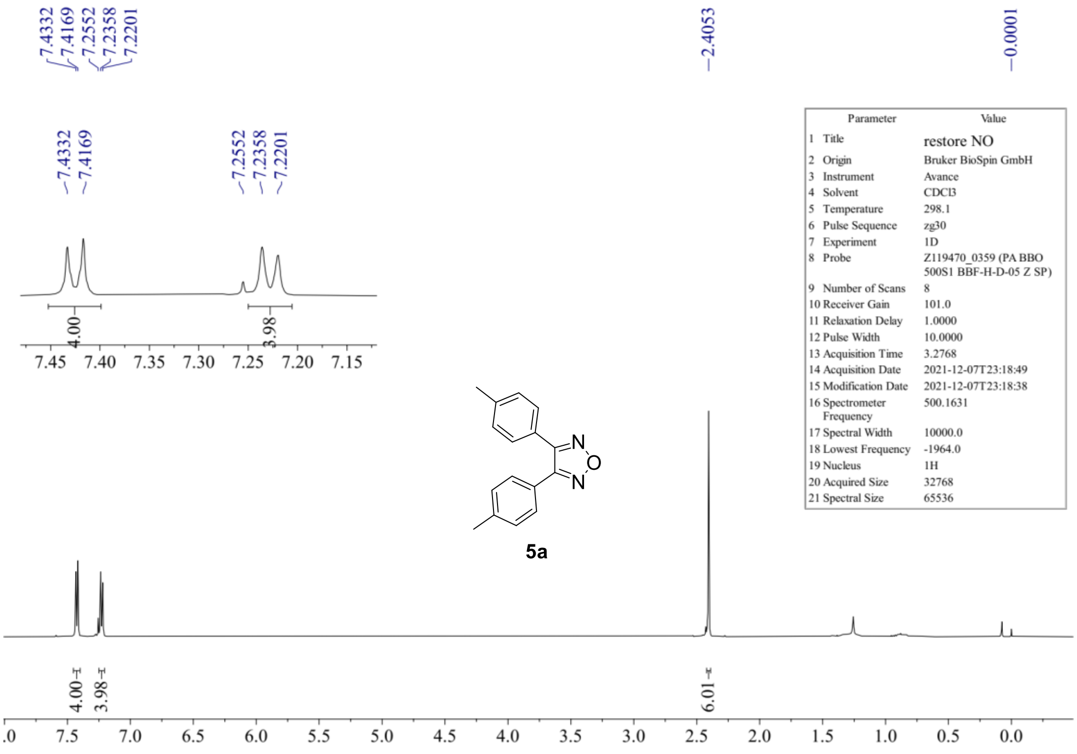

Figure S46. ^{1}H NMR (500 MHz, $CDCl_3$) of compound **5a**.

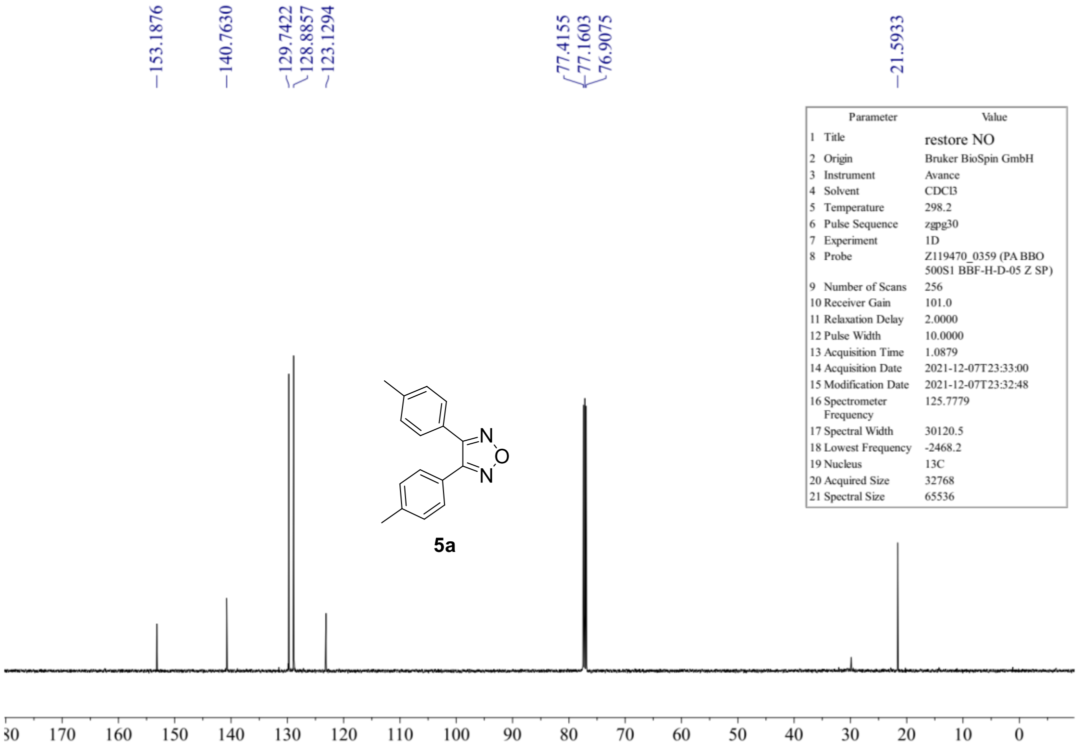

Figure S47. ^{13}C NMR (126 MHz, $CDCl_3$) of compound **5a**.

3. X-Ray single-crystal data of compound 2c.

Single crystals of **2c** were obtained by slow evaporation from a mixture of dichloromethane/*n*-hexane at 4 ˚C. Single-crystal X-ray diffraction data were collected on a diffractometer (Gemini S Ultra, Agilent Technologies) equipped with a CCD area detector using graphite-monochromated Cu*Kα* radiation (λ = 1.54184 Å) in the scan range 9.438° < 2θ < 133.12°. The structure was solved with direct methods using SHELXT and refined with full-matrix least-squares refinement using the SHELXL program within OLEX2. Crystallographic data have been deposited in the Cambridge Crystallographic Data Centre as deposition number CCDC 2157590.

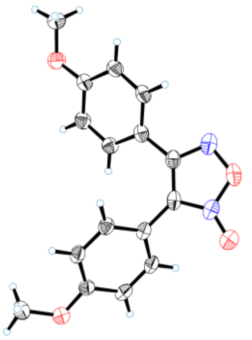

Figure S48. ORTEP diagram of **2c** with 30% thermal ellipsoids

Table S1. Crystal data for **2c**.

Identification code	2157590
Empirical formula	$C_{16}H_{14}N_2O_4$
Formula weight	298.29
Temperature/K	293(2)
Crystal system	orthorhombic
Space group	Pbca
a/Å	12.2790(4)
b/Å	8.1025(2)
c/Å	29.0019(8)
α/°	90
β/°	90
γ/°	90
Volume/Å^3	2885.42(14)
Z	8
ρ_{calc}g/cm^3	1.373
μ/mm^{-1}	0.834
F(000)	1248.0
Crystal size/mm^3	0.22 × 0.15 × 0.08
Radiation	CuKα (λ = 1.54184)
2θ range for data collection/°	9.438 to 133.12
Index ranges	$-14 \leq h \leq 14$, $-9 \leq k \leq 7$, $-22 \leq l \leq 34$
Reflections collected	6400
Independent reflections	2545 [Rint = 0.0211, Rsigma = 0.0153]
Data/restraints/parameters	2545/1/202
Goodness-of-fit on F^2	1.060
Final R indexes [I>=2σ (I)]	R_1 = 0.0721, wR_2 = 0.2063
Final R indexes [all data]	R_1 = 0.0796, wR_2 = 0.2151
Largest diff. peak/hole/e Å^{-3}	0.80/-0.41

4. References

1. Sheldon, R.A. Organic synthesis-past, present and future. *Chem. Ind.* **1992**, *23*, 903−906.
2. Sheldon, R.A. The E Factor: fifteen years on. *Green Chem.* **2007**, *9*, 1273–1283. https://doi.org/10.1039/b713736m.
3. Sheldon, R.A. The E factor 25 years on: the rise of green chemistry and sustainability. *Green Chem.* **2017**, *19*, 18–43. https://doi.org/10.1039/c6gc02157c.
4. Roschangar, F.; Sheldon, R.A.; Senanayake, C.H. Overcoming barriers to green chemistry in the pharmaceutical industry – the Green Aspiration Level™ concept. *Green Chem.* **2014**, *17*, 752–768. https://doi.org/10.1039/c4gc01563k.
5. Trost, B. The Atom Economy—A Search for Synthetic Efficiency. *Science* **1991**, *254*, 1471–1477. https://doi.org/10.1126/science.1962206.
6. Curzons, A.D.; Mortimer, D.N.; Constable, D.J.C.; Cunningham, V.L. So you think your process is green, how do you know?—Using principles of sustainability to determine what is green—A corporate perspective. *Green Chem.* **2001**, *3*, 1–6. https://doi.org/10.1039/b007871i.
